# A combinatorial code of transcription factors specifies subtypes of visual motion-sensing neurons in *Drosophila*

**DOI:** 10.1242/dev.186296

**Published:** 2020-05-13

**Authors:** Nikolai Hörmann, Tabea Schilling, Aicha Haji Ali, Etienne Serbe, Christian Mayer, Alexander Borst, Jesús Pujol-Martí

**Affiliations:** 1 Department of Circuits – Computation – Models, Max Planck Institute of Neurobiology, 82152 Martinsried, Germany; 2 Laboratory of Neurogenomics, Max Planck Institute of Neurobiology, 82152 Martinsried, Germany

**Keywords:** *Drosophila*, Dendrite development, Motion vision, Neuronal subtypes, Combinatorial code, Grain

## Abstract

Direction-selective T4/T5 neurons exist in four subtypes, each tuned to visual motion along one of the four cardinal directions. Along with their directional tuning, neurons of each T4/T5 subtype orient their dendrites and project their axons in a subtype-specific manner. Directional tuning, thus, appears strictly linked to morphology in T4/T5 neurons. How the four T4/T5 subtypes acquire their distinct morphologies during development remains largely unknown. Here, we investigated when and how the dendrites of the four T4/T5 subtypes acquire their specific orientations, and profiled the transcriptomes of all T4/T5 neurons during this process. This revealed a simple and stable combinatorial code of transcription factors defining the four T4/T5 subtypes during their development. Changing the combination of transcription factors of specific T4/T5 subtypes resulted in predictable and complete conversions of subtype-specific properties, i.e. dendrite orientation and matching axon projection pattern. Therefore, a combinatorial code of transcription factors coordinates the development of dendrite and axon morphologies to generate anatomical specializations that differentiate subtypes of T4/T5 motion-sensing neurons.

## Abstract (precis)

**Summary:** Morphological and transcriptomic analyses allowed the identification of a combinatorial code of transcription factors that controls the development of subtype-specific morphologies in motion-detecting neurons of the *Drosophila* visual system.

## INTRODUCTION

A central question in developmental neuroscience is how different neuronal cell types acquire the diverse morphologies and connectivities that support their distinct functions within complex neural circuits. The T4/T5 neuronal population of the *Drosophila* visual system provides a unique model for the study of this process. All T4/T5 neurons must acquire common morphological properties that set them apart from other visual interneurons and are important for their function as local motion sensors (Maisak et al., 2013; Shinomiya et al., 2015; Schilling et al., 2019). However, among the T4/T5 neurons, distinct subtypes with anatomical specializations relevant for the detection of motion along different directions must be specified (Fischbach and Dittrich, 1989; Maisak et al., 2013). Here, we examine the genetic programmes that control the development of subtype-specific morphologies in postmitotic T4/T5 neurons.

In *Drosophila*, visual information from ∼800 retinal ommatidia is processed in distinct neuropils (lamina, medulla, lobula and lobula plate), each consisting of retinotopically arranged columns. All neuropils except the lamina are further divided into synaptic layers (Fischbach and Dittrich, 1989; Bausenwein et al., 1992). The dendrites of T4 and T5 neurons are the first stage within the visual processing pathway in which directional motion information is extracted (Maisak et al., 2013; Behnia et al., 2014; Fisher et al., 2015; Serbe et al., 2016; Arenz et al., 2017). T4 dendrites arborise in layer 10 of the medulla and selectively respond to ON (bright edge) motion, whereas T5 dendrites arborise in layer 1 of the lobula and only respond to OFF (dark edge) motion (Maisak et al., 2013).

**Fig. 1.**
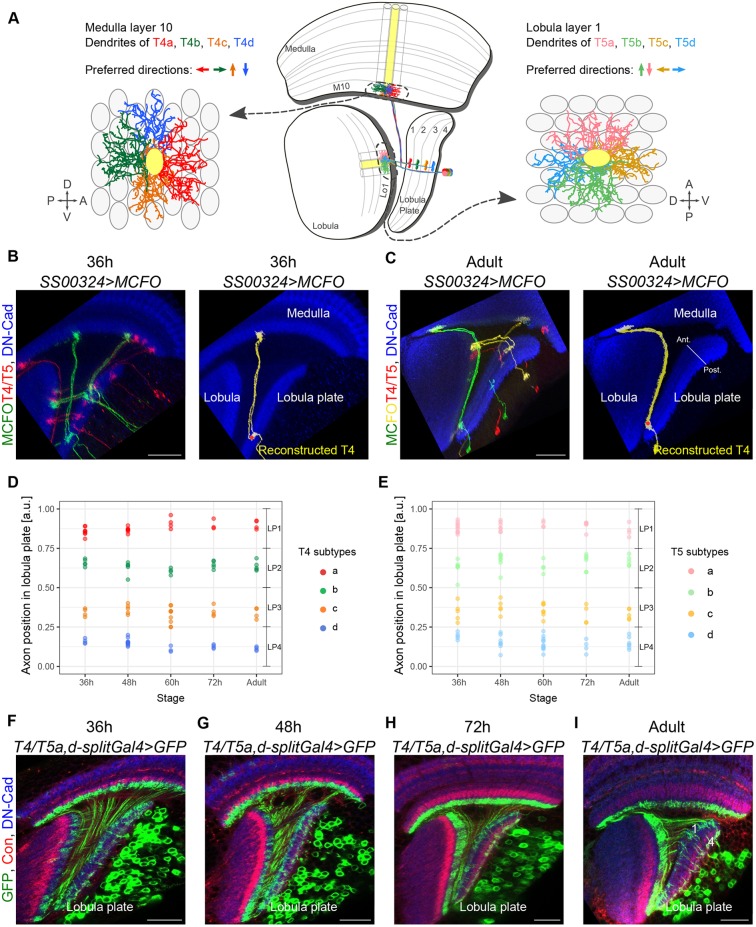
**Developing T4 and T5 subtypes can be identified by the positions of their axons in the lobula plate.** (A) Schematic of adult optic lobe (horizontal view, central panel) highlighting the morphologies of the four T4/T5 subtypes (a, b, c and d). Each of the four lobula plate layers (1-4) receives axons from only one T4/T5 subtype. Left and right panels show frontal views of medulla layer 10 and lobula layer 1. Four T4 and T5 dendrites (each of one subtype) enter a single medulla and lobula column (in yellow) to extend along four distinct directions. Arrows indicate the dendrites' preferred directions of motion. A, P, D and V: anterior, posterior, dorsal and ventral (visual field coordinates). (B,C) Optic lobes at 36 h APF and at the adult stage showing individual T4 and T5 neurons labelled with different fluorescent proteins using the MCFO approach. A digitally reconstructed T4 neuron is shown for each of the stages. The red dot marks the axon's first branching point, which was used to calculate the relative position in the lobula plate occupied by the axon. Anti-DN-Cadherin (DN-Cad) labels the neuropils. (D,E) Relative positions in the lobula plate occupied by axons of single T4 (*n*=104) and T5 (*n*=122) neurons at different developmental stages (36-72 h APF), and at the adult stage. LP1-4 refers to the regions that correspond to the lobula plate layers 1-4 at the adult stage. Each T4 and T5 neuron was classified into one of the four subtypes based on the position of the lobula plate occupied by its axon. a.u., arbitrary units. (F-I) At the adult stage, the *T4/T5a*,*d-splitGal4* driver line labels T4/T5a,d neurons with axons innervating lobula plate layers 1 and 4 (I). From 36 to 72 h APF (F-H), this line labels T4/T5 neurons with axons in lobula plate regions that correspond to the lobula plate layers 1 and 4 at the adult stage. Anti-Connectin (Con) labels layers 3 and 4 of the lobula plate. Scale bars: 20 µm.

Each T4 and T5 dendrite extends across approximately eight neuropil columns to receive signals from various presynaptic partners that relay information from neighbouring points in the visual space (Haag et al., 2016; Shinomiya et al., 2019). Both T4 and T5 neurons exist in four subtypes of equal numbers (a, b, c and d) (Pinto-Teixeira et al., 2018), each with the dendrite oriented preferentially along one of four directions within the respective neuropil (Takemura et al., 2013). In accordance with their distinct dendrite morphologies, the four T4/T5 subtypes respond to either front-to-back, back-to-front, upward or downward motion (Maisak et al., 2013). Therefore, the directional tunings of the four T4/T5 subtypes appear to be strictly linked to their dendrite orientations (Fig. 1A). In addition, the four T4/T5 subtypes exhibit distinct axon projection patterns. Axons from T4/T5 neurons of the same subtype exclusively innervate one of the four lobula plate layers (Fig. 1A) (Fischbach and Dittrich, 1989; Shinomiya et al., 2019). The segregation of T4/T5 axons into four layers, each encoding motion in a different cardinal direction, provides the anatomical basis for subsequent processing steps performed by downstream neurons that are relevant for motion-driven behaviours, e.g. the integration of opposing motions in the visual field (Mauss et al., 2015; Klapoetke et al., 2017).

Recent studies have uncovered the developmental genetic programmes that take place in T4/T5 neuron progenitors to specify T4/T5 neurons into the four subtypes (Apitz and Salecker, 2018; Pinto-Teixeira et al., 2018). During the differentiation of postmitotic T4/T5 neurons, these programmes must be translated into the expression of effector genes ensuring that four subgroups of T4/T5 neurons develop dendrites oriented along four different directions in common extracellular environments. In addition, the development of a specific dendrite orientation must be strictly coupled to the placement of the axon terminal in a specific lobula plate layer in order to relay specific qualities of directional motion to correct downstream neurons (Fig. 1A). Until now, only one gene [*optomotor-blind* (*omb*); also known as *bifid*] has been proposed to act in differentiating T4/T5c and T4/T5d to distinguish their axons from those of T4/T5a and T4/T5b neurons (Apitz and Salecker, 2018). Therefore, the following questions have remained elusive so far: (1) how do axons of T4/T5a and T4/T5b or axons of T4/T5c and T4/T5d become distinct from each other?; (2) how do the four T4/T5 subtypes acquire their four different dendrite orientations?; and (3) how is dendrite orientation matched to axon projection layer within each subtype?

Here, we first analysed the dendrite growth patterns of the four T4/T5 subtypes. The dendrites of all T4/T5 subtypes grow simultaneously during a ∼36 h-window of pupal development to acquire the oriented arbours that define their adult morphology. To investigate the underlying molecular mechanisms, we used single cell RNA sequencing (scRNA-seq) to profile the transcriptomes of T4/T5 subtypes at five stages that cover the period of dendrite growth. Our analysis revealed that each T4/T5 subtype is defined by a unique combination of cell-membrane proteins, as well as by a unique combination of two to three transcription factors that is stable for most of the dendrite growth period. To test whether such transcription factor combinations control the development of subtype-specific dendrite orientations, we manipulated them in specific T4/T5 subtypes. Overexpressing the transcription factor Grain (normally expressed only in T4/T5b and T4/T5c neurons) in all developing T4/T5 subtypes resulted in neurons with dendrite orientations specific to either T4/T5b or T4/T5c subtypes. Therefore, Grain is sufficient to invert the orientation of developing dendrites in T4/T5a and T4/T5d subtypes to generate dendrites typical of T4/T5b and T4/T5c subtypes, respectively. In addition, *grain*-overexpressing neurons with T4/T5b and T4/T5c dendrite orientations also project their axons to layers of the lobula plate normally innervated by T4/T5b and T4/T5c subtypes, respectively. Conversely, *grain* loss of function in all developing T4/T5 neurons resulted in neurons with morphologies characteristic of either T4/T5a or T4/T5d subtypes. We conclude that Grain, in combination with subtype-specific sets of transcription factors, coordinates dendrite and axon development in T4/T5b and T4/T5c to differentiate their morphologies from those of T4/T5a and T4/T5d.

## RESULTS

### Directed dendrite growth of the four T4 and T5 neuron subtypes occurs simultaneously

We first sought to investigate when and how each T4/T5 subtype acquires its defining dendrite orientation. We stochastically labelled individual T4 and T5 neurons with different combinations of fluorescent proteins using the MultiColor FlpOut (MCFO) approach (Nern et al., 2015) together with the *SS00324-splitGal4* line that drives expression specifically in all T4/T5 neurons (Schilling and Borst, 2015). This allowed us to digitally reconstruct a total of 226 T4 and T5 neurons at four stages of pupal development [36, 48, 60 and 72 h after puparium formation (APF)] and in adult flies (Fig. 1B,C). After measuring the positions within the lobula plate in which the axon terminals of adult T4 and T5 neurons enter, we found four clusters of T4 and four clusters of T5 neurons (Fig. 1D,E). These clusters represent the four T4 and T5 subtypes (a, b, c and d), with axons innervating the four lobula plate layers and with four distinct dendrite orientations (Fig. 1A) (Fischbach and Dittrich, 1989; Takemura et al., 2013). Similarly, four axon-position-based clusters of T4 and T5 neurons were found in every examined developmental stage (Fig. 1D,E). Once established, the positions occupied by T4 and T5 axon terminals in the lobula plate did not appear to change, as a driver line labelling T4/T5 neurons with axons in layers 1 and 4 of the lobula plate at the adult stage also labelled T4/T5 neurons innervating the corresponding regions of the lobula plate at earlier stages of development (Fig. 1F-I). Therefore, the T4 and the T5 subtypes can be reliably identified from 36 h APF onwards by the position of their axons in the lobula plate.

**Fig. 2.**
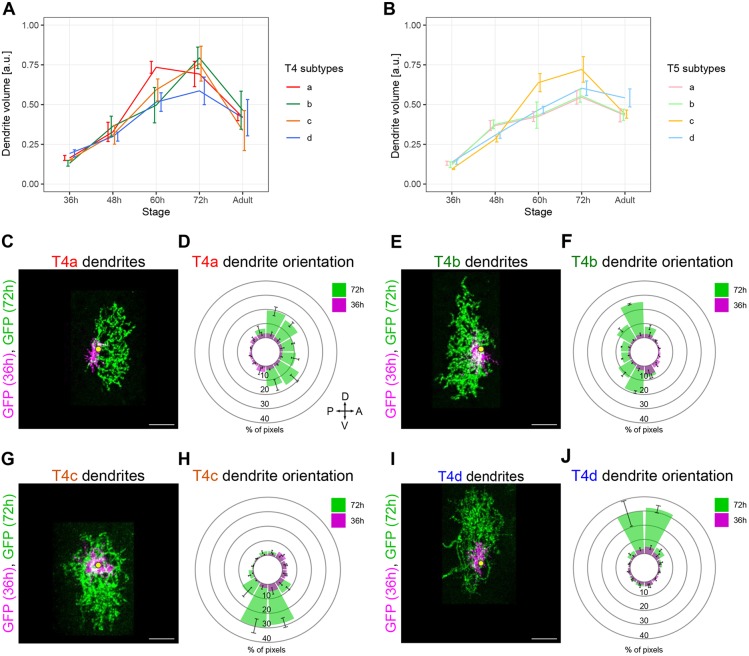
**Directed dendrite growth of the four T4 and T5 subtypes occurs simultaneously.** (A,B) Volumes from T4 (*n*=104) and T5 (*n*=122) dendrites of the four subtypes at different developmental stages, and at the adult stage. Data are mean±s.e.m. a.u., arbitrary units. (C,E,G,I) Overlay of two different T4a, T4b, T4c or T4d dendrites imaged at 36 and 72 h APF. Yellow dots mark the dendrite′s first branching point. Scale bars: 5 µm. (D,F,H,J) Average dendrite orientation of the four T4 subtypes at 36 and 72 h APF (*n*=4 per subtype and stage). Polar histograms show the 2D distribution of fluorescent pixels around the dendrite's first branching point. The number of pixels at 36 h APF was normalised to the number of pixels at 72 h APF to visualise dendrite size changes. Data are mean±s.e.m.

Next, we measured the dendrite volume of every reconstructed T4/T5 neuron and examined changes during development in the different T4/T5 subtypes. The four T4/T5 subtypes grew their dendrites at similar rates between 36 and 72 h APF. Afterwards, between 72 h APF and the adult stage, all T4/T5 dendrites underwent a reduction in volume (Fig. 2A,B). Two different mechanisms to develop oriented dendrites are compatible with these observations: (1) T4/T5 dendrites might undergo a symmetrical overgrowth of branches towards all directions (36-72 h APF) followed by a period in which branches with wrong orientations are eliminated (72 h APF-adult stage); or, alternatively, (2) the dendritic branches of each T4/T5 neuron might grow in specific directions during the period of dendrite growth (36-72 h APF). To distinguish between these possibilities, we examined the dendrite orientation of developing T4 neurons by quantifying the 2D distribution of branches around the dendrite's first branching point. Adult T4 dendrites, either imaged by confocal microscopy or reconstructed from electron microscopy data (Takemura et al., 2017), showed subtype-specific dendrite orientations that fitted with those originally reported (Takemura et al., 2013) (Fig. S1). The quantification of T4 dendrite orientations at 36 and 72 h APF revealed that subtype-specific orientations arose between those two developmental stages (Fig. 2C-J). Collectively, these results indicate that the four T4/T5 subtypes acquire their characteristic dendrite orientations through simultaneous processes of directed growth that span a ∼36 h window of development, and that subsequent dendrite pruning does not play a major role in shaping dendrite orientation.

### Each of the four T4 and T5 subtypes has a unique transcriptional profile during dendrite growth

The dendrites of the four T4 subtypes grow simultaneously within layer 10 of the medulla, and thus they share a common extracellular environment. The same holds true for the dendrites of the four T5 subtypes in layer 1 of the lobula. We hypothesised that, in order to develop different dendrite orientations, the four T4/T5 subtypes must rely on intrinsic molecular asymmetries such that their dendrites respond differentially to extrinsic cues available to all of them. Recent studies have profiled the transcriptomes of T4/T5 neurons at the adult stage. These studies either were not suitable for the analysis of T4/T5 subtype-specific transcriptomes (Pankova and Borst, 2016; Davie et al., 2018; Konstantinides et al., 2018) or explored gene expression differences only between two subtype-pairs at the adult stage (Davis et al., 2020), likely missing genes underlying the development of the morphologies defining the four T4/T5 subtypes.

**Fig. 3.**
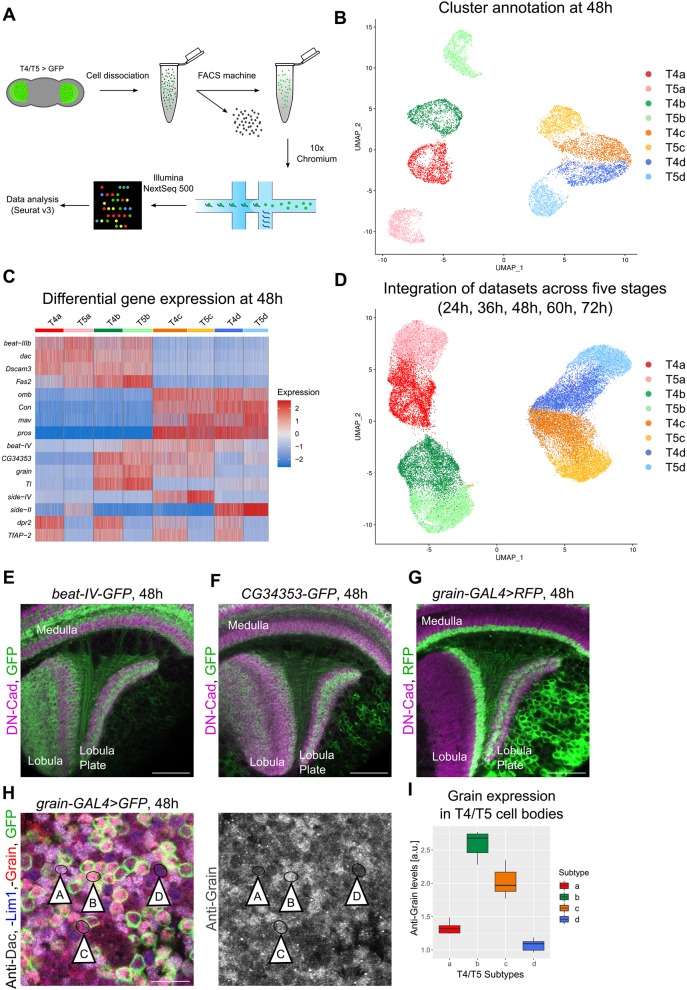
**Each T4 and T5 subtype has a unique transcriptional profile during dendrite growth.** (A) Schematic of scRNA-seq protocol. (B) Visualization of T4/T5 neurons sequenced at 48 h APF using UMAP after dimensionality reduction by PCA and unsupervised clustering based on the Louvain algorithm. Dots represent single cells and are arranged according to transcriptome similarity. We assigned clusters to either T4 or T5 based on *TfAP-2* expression, and to T4/T5a,b, T4/T5c,d or T4/T5b,c based on *dac*, *omb* or *grain* expression. (C) Heat map showing the expression levels of the 16 genes differentially expressed between the single cell clusters of T4 and T5 subtypes found in every developmental stage. Columns represent cells and were grouped based on cluster identities. Genes (rows) were manually ordered based on similarity of subtype-specific expression patterns. (D) Integration of scRNA-seq datasets across all developmental stages (24, 36, 48, 60, 72 h APF). Cells were previously assigned to four T4 and T5 subtypes at each developmental stage as described in B. (E-G) At 48 h APF, *beat-IV-GFP* and *CG34353-GFP* MiMIC lines (endogenous GFP-tagging of proteins), and the *grain-Gal4* driver line label specifically T4/T5 neurons with axons in the regions of the lobula plate corresponding to adult lobula plate layers 2 and 3 (innervated by T4/T5b,c neurons). (H) Anti-Lim1 and Anti-Dac immunostainings mark T4/T5a-d and T4/T5a,b cell bodies, respectively. *grain-Gal4* labels T4/T5b,c neurons. The combination of these markers allowed the identification of T4/T5 cell bodies of the four subtypes at 48 h APF (arrowheads, A, B, C and D). Anti-Grain immunostaining signal is enriched specifically in cell bodies of T4/T5b,c (*grain-Gal4^+^*). (I) Quantification of anti-Grain immunostaining in T4/T5 cell bodies of the four subtypes at 48 h APF supports that *grain* is specifically expressed in T4/T5b,c neurons (*n*=4 optic lobes). The end of the whiskers represent the minimum and maximum values. a.u., arbitrary units. Scale bars: 20 µm (E-G); 10 µm (H).

To overcome these limitations, we profiled the transcriptomes of single T4/T5 neurons collected at four equally spaced developmental stages during dendrite growth (36, 48, 60 and 72 h APF), as well as a preceding stage (24 h APF). For each stage, we dissected brains containing all T4/T5 neurons labelled by membrane-targeted GFP expressed by the line *SS00324-splitGal4*. Single cell suspensions were prepared and GFP^+^ T4 and T5 cells were sorted by fluorescence-activated cell sorting (FACS). Next, we performed scRNA-seq based on droplet microfluidics (10x Chromium) (Fig. 3A). Cells were sequenced to a mean depth of 26,153 reads per cell, and a median of 1627 genes were detected per cell. After filtering to remove low-quality cells, we obtained the transcriptomes of ∼44 K high-quality cells, with the number of cells per stage ranging between 5051 (60 h APF) and 11,716 (72 h APF). Two biological replicates were obtained for each developmental stage and batch-corrected using canonical correlation analysis in Seurat v3 (Stuart et al., 2019). Next, we implemented dimensionality reduction and unsupervised clustering methods based on principal component analysis (PCA) and the Louvain algorithm (Seurat v3). For each developmental stage, we manually assigned clusters either to T4 or T5 types based on known marker genes such as *TfAP-2* (Davis et al., 2020). We found that four clusters can be grouped reliably into each type (Fig. 3B; Fig. S2).

Based on the previously reported subtype-specific marker genes *omb* and *dachshund* (*dac*) (Apitz and Salecker, 2018), we assigned clusters to one of the following subtype-pairs: T4a,b; T5a,b; T4c,d; or T5c,d (Fig. 3B; Fig. S2). To identify novel marker genes discriminating the clusters within each pair, we performed a differential gene-expression analysis (Fig. 3C; Fig. S2). The results revealed that one cluster from each pair consistently showed differential co-expression of *beat-IV*, *CG34353* and *grain.* We examined the expression patterns of these genes *in vivo* with transgenic lines and antibody staining and found that they constituted specific markers of T4/T5b and T4/T5c neurons (Fig. 3E-I). Taken together, the use of three known and three newly characterised T4/T5 neuron subtype-specific marker genes was sufficient to assign all eight single cell clusters to four T4 and four T5 subtypes in every examined developmental stage (Fig. 3B; Fig. S2). Consistently, the integration of scRNA-seq datasets across all developmental stages, using the integration tool from Seurat v3, grouped all cell types in agreement with our manual cluster assignment at each stage (Fig. 3D).

### Analysis of gene expression patterns reveals combinatorial codes potentially controlling the development of the four T4/T5 dendrite orientations

**Fig. 4.**
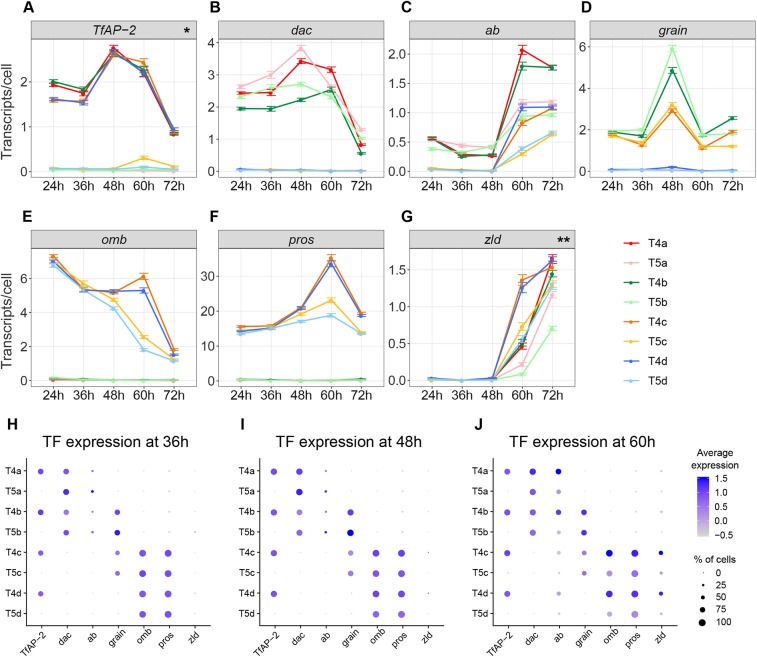
**A combinatorial code of transcription factors defines the four T4/T5 subtypes during dendrite growth.** (A-G) Subtype-specific expression patterns and dynamics of transcription factors that are differentially expressed between subtypes of T4/T5 neurons. *y*-axis shows the count of transcripts per cell (mean± s.e.m.). *x*-axis shows developmental stage (h APF). *Higher expression in all T4 subtypes than in all T5 subtypes. **Differential expression between T4/T5 subtypes only during the last phase of dendrite growth (60-72 h APF). (H-J) Dot plots showing the mean scaled expression levels (colour-coded) of each transcription factor (TF) in the different T4/T5 subtypes at 36, 48 and 60 h APF. Dot sizes represent the percentage of cells in which the transcription factor was detected. Transcription factors were manually ordered based on the similarity of subtype-specific expression patterns.

Transcription factors act as intrinsic determinants of dendrite shape, in part by controlling the expression of cell-membrane proteins relevant for sensing extrinsic cues (Puram and Bonni, 2013; Dong et al., 2015; Lefebvre et al., 2015; Prigge and Kay, 2018). The discovery of transcriptionally different groups of T4/T5 neurons that match morphologically distinct T4/T5 subtypes during dendrite growth allowed us to search for candidate genes that control subtype-specific dendrite orientations. To identify differentially expressed genes, we ran differential expression tests separately for each developmental dataset. We required genes to have a twofold change to be considered differentially expressed. We found seven genes encoding for transcription factors and 62 genes encoding for cell-membrane proteins (excluding neurotransmitter/neuropeptide receptors, ion channels and transporters) that were differentially expressed between the transcriptionally distinct groups of T4/T5 neurons at any of the examined stages. Further analysis of gene expression patterns revealed that 22 out of the 69 genes (32%) had either higher expression levels in all T4 subtypes than in all T5 subtypes, or vice versa, at some point during development (e.g. *TfAP-2* and *CG14340*) or with subtype-specific expression patterns only in T4 or T5 neurons (e.g. *dpr3* and *DIP-Θ*) (Fig. 4A; Fig. S3A). We hypothesised that these genes probably play a role in defining properties of T4 versus T5 neurons.

The other 47 out of the 69 genes (68%) exhibited subtype-specific expression patterns and dynamics that were remarkably similar between T4 and T5 neurons (Fig. 4B-G; Fig. S3A), thus positing them as candidates for controlling subtype-specific traits shared between T4 and T5 neurons, like the four dendrite orientations. We attempted to narrow down this list of 47 candidate genes by further exploring their expression dynamics. We found six genes differentially expressed between T4/T5 subtypes only during the last phase of dendrite growth (60-72 h APF) (e.g. *zld* and *fz2*) (Fig. 4G, Fig. S3A). This period coincides with the onset of synaptogenesis in the *Drosophila* central nervous system (Chen et al., 2014; Muthukumar et al., 2014), suggesting an involvement of these genes in this process. Another ten genes exhibited subtype-specific expression patterns that switched over time (e.g. *kuz* and *Lac*) (Fig. S3A). Because such discontinuous and/or late subtype-specific expression patterns during dendrite growth are unlikely to contribute to the development of four dendrite orientations, we discarded these genes.

The resulting list of 31 candidate genes potentially controlling the development of the four dendrite orientations contained only one gene that was exclusively expressed in a single T4/T5 subtype (*side-IV*) (Fig. S3A,B). Within the remaining genes, some genes were clearly co-expressed in several subtypes, although not necessarily at the same levels. For example, we found genes specific to T4/T5a,b (*Dscam3*), T4/T5c,d (*robo3*), T4/T5b,d (*Tl*), T4/T5a,d (*side-II*), T4/T5b,c,d (*beat-IV*) and T4/T5a,c,d (e.g. *kek1*) (Fig. S3B). These results indicate that the four T4/T5 subtypes are defined by combinatorial codes of gene expression that might underlie the development of the four different dendrite orientations.

### Grain acts as part of two combinations of transcription factors controlling the dendrite orientations and matching axon projection patterns of two T4/T5 subtypes

Combinatorial codes of transcription factors control the development of subtype-specific traits in postmitotic neurons (Allan and Thor, 2015; Hobert and Kratsios, 2019). Only five transcription factors were present in our list of 31 candidate genes potentially controlling the development of the four T4/T5 dendrite orientations: *dac*, *omb*, *abrupt* (*ab*), *prospero* (*pros*) and *grain*. Consistent with our scRNA-seq analysis, a previous study found that *dac* and *omb* were expressed in postmitotic developing T4/T5a,b and T4/T5c,d neurons, respectively (Fig. 4B,E) (Apitz and Salecker, 2018). Our scRNA-seq analysis further revealed that *ab* was enriched in T4/T5a,b (Fig. 4C), whereas *pros* was enriched in T4/T5c,d (Fig. 4F). Because of their expression patterns (T4/T5a,b versus T4/T5c,d), the combination of these four transcription factors alone were not sufficient to divide T4/T5 neurons into four subtypes (T4/T5a-d). Interestingly, *grain* was expressed only in T4/T5b,c neurons (Fig 3; Fig. 4D). Therefore, the combination of a T4/T5a,b- (*dac* or *ab*), a T4/T5c,d- (*omb* or *pros*) and a T4/T5b,c- (*grain*) specific transcription factor represents a minimal set of genes to encode the identity of the four T4/T5 subtypes. Remarkably, the subtype-specific expression pattern of each of these transcription factors is stable for most of the period of T4/T5 dendrite growth (Fig. 4H-J). Altogether, these observations suggest that the unique and stable combination of transcription factors that defines each T4/T5 subtype during development controls its specific morphology.

**Fig. 5.**
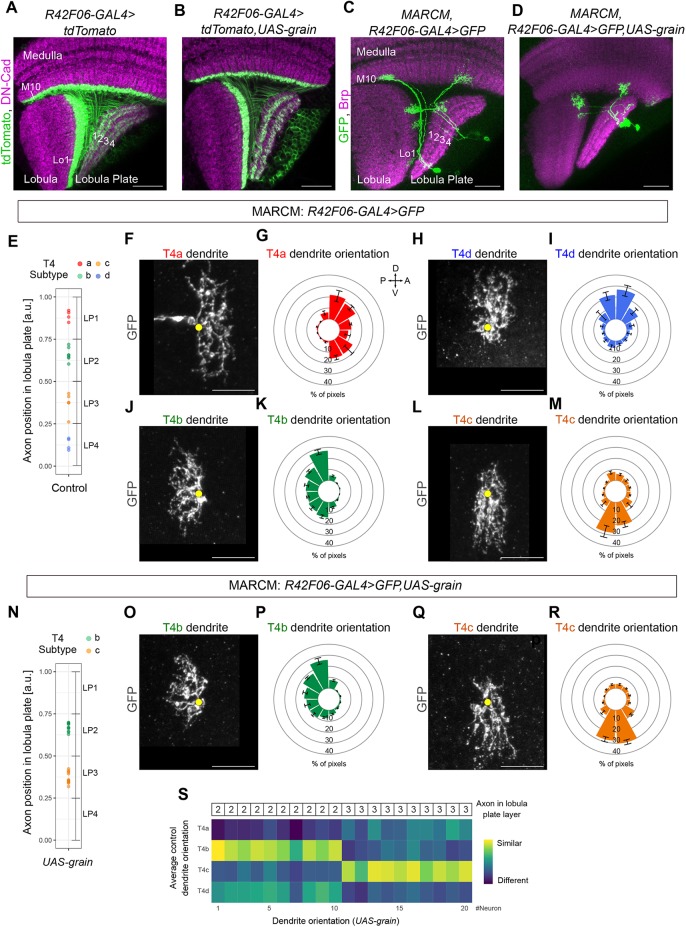
***grain* overexpression in developing T4 neurons results in adult optic lobes with only T4b,c neurons.** (A,B) Adult control T4/T5 neurons and adult T4/T5 neurons overexpressing *grain* by means of the *R42F06-Gal4* line. (C,D) Adult single-labelled T4 and T5 neurons from either control or *grain* overexpression MARCM experiments. (E) Positions in the lobula plate occupied by axon terminals of single control T4 neurons labelled by MARCM (n=20). Each T4 neuron was classified into one of the four subtypes based on its axon position in the lobula plate (T4a: *n*=4, T4b: *n*=7, T4c: *n*=5, T4d: *n*=4). (F-M) Dendrite orientations of control T4 neurons of the four subtypes classified based on axon position. Data are mean±s.e.m. (N) Positions in the lobula plate occupied by axon terminals of single, *grain*-overexpressing T4 neurons labelled by MARCM (*n*=20). *grain*-overexpressing T4 neurons project axons only to either lobula plate layer 2 (*n*=10) or lobula plate layer 3 (*n*=10). (O-R) Dendrite orientations of *grain*-overexpressing T4 neurons classified as T4b (*n*=10) or T4c (*n*=10) based on axon position. The dendrite orientations of these neurons are indistinguishable from those of wild-type T4b and T4c neurons (J-M). (S) Matrix showing colour-coded similarity indexes between the dendrite orientations of individual *grain*-overexpressing T4 neurons (*n*=20, manually ordered along the horizontal axis based on the innervated layer of the lobula plate) and the average dendrite orientations of the four control T4 subtypes (vertical axis). Yellow dots in F,H,J,L,O,Q mark the first branching point of the dendrite. a.u., arbitrary units. Data are mean±s.e.m. Scale bars: 20 µm (A-D); 5 µm (F,H,J,L,O,Q).

A prediction of this hypothesis would be that changing the code of transcription factors that a T4 or a T5 neuron expresses during development should result in a conversion of subtype-specific properties, i.e. dendrite orientations and axon projection patterns. For example, ectopic expression of *grain* in T4/T5a (normally Dac^+^/Ab^+^/Grain^−^) and T4/T5d (normally Omb^+^/Pros^+^/Grain^−^) should result in neurons with morphological properties of T4/T5b (Dac^+^/Ab^+^/Grain^+^) and T4/T5c (Omb^+^/Pros^+^/Grain^+^) subtypes. To test this hypothesis, we overexpressed *grain* in all postmitotic developing T4/T5 neurons by means of the *R42F06-Gal4* line (Maisak et al., 2013), which drives expression in maturing T4/T5 neurons before dendrite growth and axon segregation (Fig. S4A-C). This condition generated no defects in the neuropil- and layer-specific innervation of T4 and T5 dendrites. However, two, rather than four, layers of T4/T5 axons were visible in the lobula plate (Fig. 5A,B). A recent study reported similar results using different reagents and ruled out that this anatomical defect is caused by neuronal apoptosis, and proposed that the overexpression of *grain* affects T4/T5 neurons such that their axons cannot segregate to form four layers without affecting their dendrites (Kurmangaliyev et al., 2019). Alternatively, changes in T4/T5 axon projection patterns upon *grain* overexpression might result from an identity conversion of T4/T5a,d into T4/T5b,c neurons. To differentiate between these possibilities, we overexpressed *grain* in individual developing T4 and T5 neurons of all subtypes and labelled them by means of mosaic analysis with a repressible cellular marker (MARCM) and *R42F06-Gal4* (Fig. 5C,D). In control MARCM experiments, T4 and T5 neurons of all subtypes (axons in four lobula plate layers and four dendrite orientations) were found (Fig. 5E-M; Fig. S5A-C). By contrast, in *grain* overexpression MARCM experiments, we only found T4 and T5 neurons with axons in either lobula plate layer 2 or 3, which are normally innervated by T4/T5b or T4/T5c subtypes, respectively (Fig. 5N; Fig. S5D). Remarkably, *grain*-overexpressing T4 and T5 neurons that innervated either lobula plate layer 2 or 3 showed corresponding dendrite orientations of T4/T5b or T4/T5c subtypes (Fig. 5O-S; Fig. S5E,F). In addition, T4 and T5 neurons overexpressing *grain* did not show defects in morphological properties that are common to all T4/T5 subtypes, i.e. the restriction of dendrites and axons to single neuropil layers (Fig. 5; Fig. S5).

Three lines of evidence ruled out the possibility that T4/T5a,d-selective death might cause the presence of exclusively T4/T5b,c neurons in the adult upon *grain* overexpression. First, we found no difference in the number of single-labelled T4 and T5 neurons between control and *grain* overexpression MARCM experiments (Fig. S6A). Second, a single neuroblast precursor of T4/T5 neurons always produces four neurons, either T4a/T5a/T4b/T5b or T4c/T5c/T4d/T5d, that project to the same retinotopic position (Fig. S6B) (Pinto-Teixeira et al., 2018). In MARCM experiments with *grain* overexpression, we also found clones of four T4/T5 neurons projecting to the same retinotopic position, and thus originating from the same neuroblast. However, these clones consisted of either T4b/T5b/T4b/T5b or T4c/T5c/T4c/T5c neurons (*n*=3/3 clones of four T4/T5 neurons) (Fig. S6C). Third, *grain* overexpression with the *T5d-splitGal4* line, which drives expression in T5d neurons before dendrite growth and axon segregation (Fig. S4D; Fig. S6D), produced changes in axon projection patterns consistent with T5d transformation into T5c neurons (Fig. S6E). These experiments demonstrate that *grain* overexpression in developing T4/T5a and T4/T5d neurons transforms them into T4/T5b and T4/T5c neurons, respectively, based on their dendrite orientations and axon projection patterns.

**Fig. 6.**
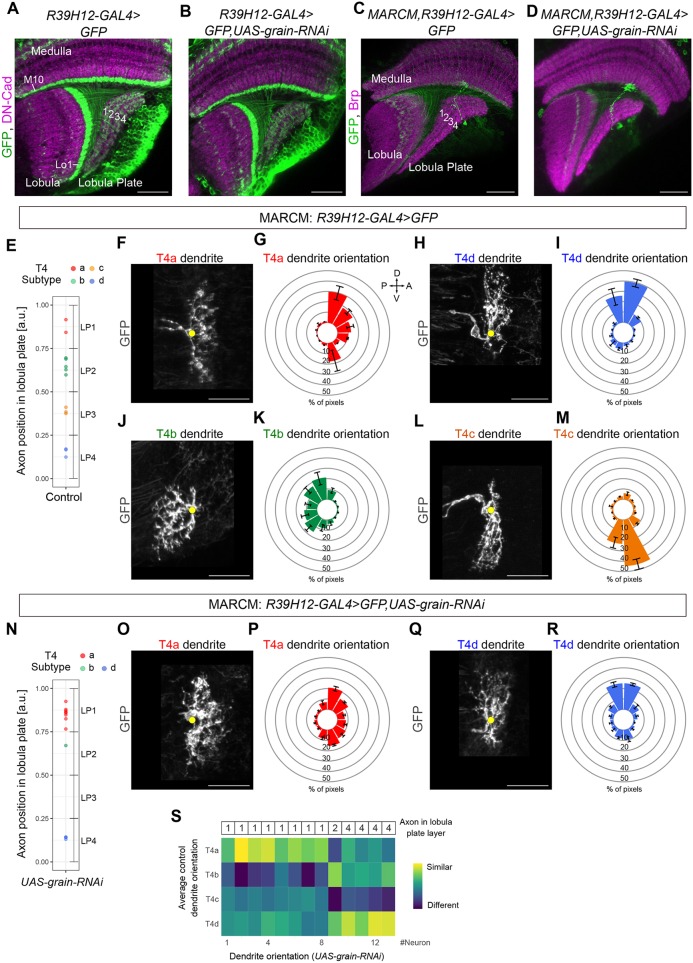
***grain* loss of function in developing T4 neurons results in adult optic lobes with mainly** **T4a,d neurons.** (A,B) Adult control T4/T5 neurons and adult T4/T5 neurons expressing *grain-RNAi* with the *R39H12-Gal4* line. (C,D) Adult single-labelled T4 neurons from either control or *grain-RNAi* MARCM experiments. (E) Positions in the lobula plate occupied by axon terminals of single, control T4 neurons labelled by MARCM (*n*=13). Each T4 neuron was classified into one of the four subtypes based on its axon position in the lobula plate (T4a: *n*=2, T4b: *n*=5, T4c: *n*=3, T4d: *n*=3). (F-M) Dendrite orientations of control T4 neurons of the four subtypes classified based on axon position. Data are mean±s.e.m.. (N) Positions in the lobula plate occupied by axon terminals of single T4 neurons expressing *grain-RNAi* and labelled by MARCM (*n*=13). Most T4 neurons with *grain* knockdown project axons to either lobula plate layer 1 (*n*=8) or lobula plate layer 4 (*n*=4). (O-R) Dendrite orientations of T4 neurons with *grain* knockdown classified as T4a (*n*=8) or T4d (*n*=4) based on axon position. The dendrite orientations of these neurons are indistinguishable from those of wild-type T4a and T4d neurons (F-I). (S) Matrix showing colour-coded similarity indexes between the dendrite orientations of individual T4 neurons expressing *grain-RNAi* (*n*=13, manually ordered along the horizontal axis based on the innervated layer of the lobula plate) and the average dendrite orientations of the four control T4 subtypes (vertical axis). Yellow dots in F,H,J,L,O,Q mark the first branching point of the dendrite. a.u., arbitrary units. Data are mean±s.e.m. Scale bars: 20 µm (A-D); 5 µm (F,H,J,L,O,Q).

Finally, we tested whether *grain* loss of function in T4/T5b (normally Dac^+^/Ab^+^/Grain^+^) and T4/T5c (normally Omb^+^/Pros^+^/Grain^+^) results in neurons with morphological properties of T4/T5a (Dac^+^/Ab^+^/Grain^−^) and T4/T5d (Omb^+^/Pros^+^/Grain^−^) subtypes. To this end, we first performed a knockdown of *grain* in all developing T4/T5 neurons with RNAi and the *R39H12-Gal4* line, which drives expression in T4/T5 neurons of all subtypes from the late third instar (L3) larval stage onwards (Schilling et al., 2019). This resulted in adult T4/T5 neurons with dendrites that showed no defects in their neuropil- and layer-specific innervation but with axons that failed to form four layers in the lobula plate (Fig. 6A,B). Next, we employed MARCM to express *grain-RNAi* in individual maturing T4 neurons with the *R39H12-Gal4* line and to further analyse their morphology in adult brains (Fig. 6C,D). In *grain-RNAi* MARCM experiments, most T4 neurons innervated either lobula plate layer 1 or 4 and showed dendrite orientations of T4/T5a or T4/T5d subtypes, respectively, which is consistent with a transformation of T4/T5b,c into T4/T5a,d upon *grain* loss of function (Fig. 6E-S). Collectively, our data indicate that Grain acts as part of two different combinations of transcription factors, one of them differentiating T4/T5b from T4/T5a and the other one differentiating T4/T5c from T4/T5d morphologies.

## DISCUSSION

The development of neuronal morphology relies on the interplay between cell-intrinsic factors, i.e. genetic programmes, and extracellular cues, e.g. growth factors (Sanes and Zipursky, 2010; Melnattur and Lee, 2011; Dong et al., 2015). In this study, we investigated the genetic programmes underlying the acquisition of the different morphologies defining the four T4/T5 subtypes, which are essential for detecting visual motion along the four cardinal directions. Our work reveals that the development of T4/T5 subtype-specific morphologies relies on a postmitotic combinatorial code of transcription factors. In particular, Grain acts together with different transcription factors in T4/T5b and T4/T5c subtypes to coordinate dendrite and axon morphogenesis in order to differentiate their morphologies from those of T4/T5a and T4/T5d, respectively. The coordinated regulation of different aspects of neuron morphogenesis by the same set of transcription factors might provide a general strategy to ensure the establishment of precise neuron wiring patterns during development (Enriquez et al., 2015; Santiago and Bashaw, 2017; Schilling et al., 2019). Furthermore, the combinations of transcription factors controlling the development of subtype-specific properties do not appear to regulate morphological properties that are common to all T4/T5 subtypes, i.e. the restriction of dendrites and axons to single neuropil layers. The acquisition of these morphological properties is controlled postmitotically by two transcription factors of the Sox family, SoxN and Sox102F (Contreras et al., 2018; Schilling et al., 2019). Therefore, distinct sets of transcription factors control differentially subtype-specific properties and properties that are shared by all T4/T5 subtypes, shedding light on the developmental strategies that ensure that each neuron acquires its complete morphological signature.

How do the expression patterns of T4/T5 postmitotic transcription factors arise during development? *SoxN* and *Sox102F* expression in all T4/T5 subtypes arises from temporal patterning of the neuroblasts that are precursors of T4/T5 neurons (Apitz and Salecker, 2015; Schilling et al., 2019). Spatial patterning of the neuroepithelium that generates T4/T5-producing neuroblasts results in the specific expression of *omb* in neuroblasts that are precursors of T4/T5c,d neurons. The expression of *omb* is further relayed to postmitotic developing T4/T5c,d neurons, in which it represses *dac* expression (Apitz and Salecker, 2018). In addition, each Omb^−^ neuroblast (precursor of T4/T5a,b), as well as each Omb^+^ neuroblast (precursor of T4/T5c,d), divides to produce two ganglion mother cells, only one of which has Notch activity. Only ganglion mother cells with Notch activity generate T4/T5a and T4/T5d neurons (Pinto-Teixeira et al., 2018). The transcriptional programmes downstream of this Notch-dependent fate decision remain elusive. Notch activity has been shown to repress *grain* in the aCC motoneuron of the *Drosophila* embryo (Garces and Thor, 2006). In agreement with this, *grain* is not expressed in T4/T5a,d neurons originating from ganglion mother cells with Notch activity. Together, these observations suggest that the specific expression of *grain* in postmitotic T4/T5b,c neurons could result from the Notch-dependent fate decision occurring during the final division of T4/T5-producing neuroblasts. Future studies will need to investigate how the T4/T5 subtype-specific expression of *grain*, as well as of *ab* and *pros*, is achieved during development.

Our data revealed that only one transcription factor, Grain, defines T4/T5b,c neurons during development. By contrast, T4/T5a,b and T4/T5c,d are each defined by two transcription factors: Dac and Ab are co-expressed in T4/T5a,b, whereas Omb and Pros are co-expressed in T4/T5c,d. These transcription factors with overlapping expression patterns might play redundant roles. Alternatively, they might be specialised to control different aspects of development. Systematic manipulations of the expression patterns of these transcription factors will be needed to address these possibilities, as well as to further elucidate how they act in a combinatorial manner to determine the different morphologies of the four T4/T5 neuron subtypes.

Transcription factors control dendrite growth, in part by controlling the expression of genes relevant for sensing extrinsic cues. We found many cell-membrane proteins with T4/T5 subtype-specific expression patterns that might result from the action of the combinatorial code of transcription factors that we uncovered here. In agreement with the results of a recent publication (Kurmangaliyev et al., 2019), the vast majority of cell-membrane proteins with subtype-specific expression patterns in T4 neurons exhibited the same expression patterns and dynamics in T5 neurons. These mostly included receptors, ligands, regulators of various signalling pathways, and cell-adhesion molecules, some of which have been shown to be involved in axon guidance, dendrite patterning and/or synaptic specificity in *Drosophila* (Keleman et al., 2002; Furrer et al., 2007; Zarin et al., 2014; Ward et al., 2015; Tadros et al., 2016; Li et al., 2017; Barish et al., 2018; Xu et al., 2018). We hypothesise that those cell-membrane proteins with stable subtype-specific expression patterns during, at least, the first phase of dendrite growth are the most likely candidates to regulate the development of the four dendrite orientations in a combinatorial way. However, they might also control other subtype-specific properties, e.g. axon projection patterns and connectivity with distinct postsynaptic neurons. Collectively, our data indicate that the four T4 and T5 subtypes share combinations of transcription factors and downstream effector genes that might control the development of four dendrite orientations. Yet, T4 dendrites grow in the medulla and T5 dendrites grow in the lobula. One exciting possibility is that both neuropils share extrinsic cues conveying directional information to the dendrites of T4 and T5 neurons, which might also be used as universal guideposts by other neuronal cell types that must develop oriented dendrites (Ting et al., 2014).

The dendrites of all T5 subtypes extend across the same number of neuropil columns to connect to the same set of presynaptic functionally distinct neurons signalling luminescence changes from neighbouring points in the visual space, but in a spatial order that is subtype specific. The same holds true for the dendrites of all T4 subtypes (Shinomiya et al., 2019). As a simplified example, T4a connects to Mi4 in column 1, Mi1 in column 2 and Mi9 in column 3, whereas T4b connects to Mi9 in column 1, Mi1 in column 2 and Mi4 in column 3. What could the minimal set of developmental instructions look like to ensure such a specific wiring? Interestingly, the dendrites of the four T4 and T5 subtypes all show a clear and distinct orientation with respect to the extrinsic coordinates of the neuropil that they occupy. The dendrites' intrinsic coordinates define three compartments: proximal, medial and distal. With respect to these intrinsic coordinates, the wiring of all T4 and T5 subtypes is identical. In the above example, both T4a and T4b connect to Mi4 on the proximal, to Mi1 on the medial and to Mi9 on the distal part of their dendrite. Thus, once the compartmentalization of synapses from different inputs along their dendrites is controlled by cell-intrinsic mechanisms (Lefebvre et al., 2015), the decisive point that differentiates between the subtypes is how they distinctly orient their dendrite. By growing their dendrites along different extrinsically defined directions, they could all apply the same genetic programmes to connect to a set of input neurons. This would lead to a spatial arrangement of synaptic inputs that is different for each subtype with respect to the extrinsic coordinates of the neuropil, thus supporting the detection of motion across four different directions but identical within the intrinsic coordinates of the neurons' dendrite.

We envisage that the manipulation of the genetic programmes controlling dendrite orientation in T4/T5 neurons will allow us to address these ideas systematically. Studying how the four T4/T5 neuron subtypes acquire their morphologies provides a great opportunity to link development, anatomy and function in a neuronal type that performs a computation that is conserved across visual systems (Mauss et al., 2017), which might uncover universal blueprints of neural wiring.

## MATERIALS AND METHODS

### Fly strains

Flies were raised at 25°C and 60% humidity on standard cornmeal agar medium at 12 h light/dark cycle, except for RNAi experiments, in which offspring were moved from 25°C to 29°C at late larval or early pupal stages. At pupal stages, female and male brains were analysed. At adult stages, only female brains were analysed. The following fly strains were used as driver lines: *SS00324-splitGal4* (*R59E08-AD attP40*; *R42F06-DBD attP2*) (Schilling and Borst, 2015), *T4/T5a,d-splitGal4*, *grain-Gal4* [Bloomington Drosophila Stock Center (BDSC), 42224], *R42F06-Gal4* (BDSC, 41253), *T5d-splitGal4* and *R39H12-Gal4* (BDSC, 50071). The *T4/T5a,d-splitGal4* driver line was generated by combining the *R35A10-AD* (BDSC, 70193), and *R39H12-DBD* (BDSC, 69444) hemidriver lines (Dionne et al., 2018). The *T5d-splitGal4* driver line was generated by combining the *R35A10-AD* (BDSC, 70193) and *R42H07-DBD* (BDSC, 69609) hemidriver lines. The following fly strains were used as reporter lines: *MCFO-1* (BDSC, 64085), *UAS-myr::GFP* (BDSC, 32198), *UAS-mCD8::GFP* (BDSC, 32188), *UAS-mCD8::GFP* (BDSC, 32187), *UAS-myr::tdTomato* (BDSC, 32222) and *UAS-mCD8::RFP* (BDSC, 32229). To examine the expression of *beat-IV* and *CG34353* genes *in vivo*, we used the *beat-IV-GFP* (BDSC, 66506) and *CG34353-GFP* (BDSC, 60534) MiMIC lines (Venken et al., 2011). The *UAS-grain2* line was used for *grain* overexpression experiments (a gift from J. C. G. Hombría, Universidad Pablo de Olavide, Seville, Spain) (Brown and Castelli-Gair Hombria, 2000). The *UAS-grain-RNAi* line (Vienna *Drosophila* Stock Center, shRNA-330376) was used for *grain* loss-of-function experiments. *grain* overexpression MARCM experiments were carried out by crossing virgin female *hs-Flp tub-Gal80 FRT19A*; *UAS-mCD8::GFP*; *R42F06-Gal4* (a gift from F. Pinto-Teixeira, New York University Abu Dhabi, Abu Dhabi, United Arab Emirates) to male *FRT19A*; *UAS-grain2/Sp*. *grain-RNAi* MARCM experiments were performed by crossing virgin female *hs-Flp tub-Gal80 FRT19A*; *UAS-mCD8::GFP*; *R39H12-Gal4 UAS-mCD8::GFP* to male *FRT19A*; *UAS-grain-RNAi/Sp*. L3 larvae and early pupae resulting from these crosses were heatshocked for 15-20 min in a 37°C water bath. Adult females with and without *Sp* were used as control and experimental groups, respectively.

### Antibodies and immunolabelling

The following primary antibodies were used in this study: rabbit anti-GFP (1:500, Torrey Pines Biolabs, TP401), chicken anti-GFP (1:500, Rockland, 600901215S), rabbit anti-DsRed (1:500, Clontech Laboratories, 632496), rabbit anti-HA (1:300, Cayman Chemical, 162200), rat anti-FLAG (1:200, Novus Biologicals, NBP-1-06712), chicken anti-V5 (1:500, Bethyl Laboratories, A190-118A), rat anti-DN-Cadherin (1:50, Developmental Studies Hybridoma Bank, AB528121), mouse anti-Connectin (1:50, Developmental Studies Hybridoma Bank, AB10660830), mouse anti-Bruchpilot (1:20, Developmental Studies Hybridoma Bank, AB2314866), rat anti-Elav (1:50, Developmental Studies Hybridoma Bank, Rat-Elav-7E8A10), mouse anti-Dachshund (1:20, Developmental Studies Hybridoma Bank, AB528190), rabbit anti-Lim1 (1:500, a gift from C. Desplan, New York University, New York, USA) and rat anti-Grain (1:200, a gift from A. Garcès) (Garces and Thor, 2006). Secondary antibodies used in this study were as follows (used at 1:400): Alexa Fluor 488-conjugated goat anti-rabbit (Invitrogen, A11034), Alexa Fluor 488-conjugated goat anti-chicken (Invitrogen, A10262), Alexa Fluor 488-conjugated goat anti-mouse (Thermo Fisher, A28175), Alexa Fluor 488-conjugated goat anti-rat (Invitrogen, A11006), Alexa Fluor 568-conjugated goat anti-rabbit (Life Technologies, A11011), Alexa Fluor 568-conjugated goat anti-mouse (Invitrogen, A11004), Alexa Fluor 633-conjugated goat anti-mouse (Life Technologies, A21050) and Alexa Fluor 680-conjugated goat anti-rat (Invitrogen, A21096).

For immunolabelling, brains were dissected in cold PBS and fixed in 4% paraformaldehyde (containing 0.1% Triton X-100) at room temperature for 23 min. Afterwards, they were washed three times with PBT (PBS containing 0.3% Triton X-100) and blocked with 10% normal goat serum in PBT at room temperature for 2 h. Brains were incubated with primary antibodies diluted in PBT containing 5% normal goat serum for 24-48 h at 4°C. After being washed five times with PBT, brains were incubated with secondary antibodies diluted in PBT containing 5% normal goat serum for 24-48 h at 4°C. Brains were then washed five times with PBT and once with PBS, before being mounted in SlowFade Gold Antifade Mountant (Thermo Fisher Scientific).

### Confocal imaging, and image processing and visualisation

Imaging was performed with a Leica SP8 laser scanning confocal microscope equipped with 488-, 561- and 633-nm lasers, and using a 40× or 63× objective. Deconvolution of confocal data (Figs 1,2; Fig. S1) was performed with Huygens Deconvolution software (Scientific Volume Imaging) using default parameters. Image processing and measurements were performed with the Fiji software package (Schindelin et al., 2012). Three-dimensional visualization of confocal data (Fig. S6B,C), neuron reconstructions and measurements (Figs 1,2) were performed with Amira software (Zure Institute Berlin, Thermo Fisher Scientific). Vaa3D software (Allen Institute for Brain Science) was also used for 3D visualization of confocal data (Fig. S5). All figures were prepared using Inkscape software.

In *grain-RNAi* MARCM experiments (Fig. 6), both control and experimental brains showed leaky GFP expression in most of the T4/T5 neurons. However, some brains contained single-labelled T4 and/or T5 cells expressing GFP at much higher levels than the rest of the T4/T5 neurons, which indicated the absence of the *tub-Gal80* transgene and the high expression of *UAS* transgenes in these cells. In both control and experimental brains, only T4 neurons with the highest GFP expression (showing saturated fluorescent signals with laser power of 10%, gain of 100% and pinhole of 0.6) were selected for imaging. In this way, we aimed to image and analyse only those T4 neurons with the highest expression of *UAS-grain-RNAi*. All control and experimental brains were immunolabelled and mounted in parallel following the same protocols.

### Quantification of Grain levels in T4/T5 cell bodies

Relative expression levels of Grain in T4/T5 cell bodies of different subtypes (Fig. 3H,I) were quantified as follows: For each optic lobe, we used Fiji to measure the mean fluorescence intensity (anti-Grain channel) from approximately 60 manually segmented T4 and T5 cell bodies (Lim^+^) in single optical sections. We classified each cell body into one of the four T4/T5 subtypes based on anti-Dac staining and *grain-Gal4* expression (GFP^+^). For each T4/T5 subtype, we obtained the average of Grain fluorescence per cell body and divided it by the mean fluorescence intensity (in the anti-Grain channel) of ten surrounding cell bodies that were not from T4/T5 neurons (Lim^−^). Calculations were performed using Microsoft Excel Software and plots were constructed using Python 3.6. In box-and-whisker plots, the end of the whiskers represent the minimum and maximum values.

### Morphological characterization of T4 and T5 neuron subtypes

We digitally reconstructed individual T4 and T5 neurons from deconvolved confocal image stacks (Figs 1,2) using the magic wand tool of Amira's segmentation editor, followed by surface model generation. For each neuron, the range of pixel intensities used by the magic wand tool was adjusted manually in the display and masking area. In order to classify each reconstructed T4 and T5 cell into one of the four subtypes (Fig. 1D,E), we used the relative position of the axon terminal in the lobula plate, which was quantified as follows: the distance between the axon's first branching point and the most posterior edge of the lobula plate along the anteroposterior axis was measured in a single optical section with Fiji. This value was normalised by the total length of the lobula plate along the anteroposterior axis at the proximodistal position occupied by the axon's first branching point. The numbers 0 and 1 represent the most posterior and the most anterior edges of the lobula plate, respectively. We followed a very similar procedure to classify single-labelled T4 and T5 cells in MARCM experiments (Fig. 5; Fig. S5; Fig. 6), with the only difference being that we used the position of the first axonal bouton to calculate the relative position of the axon terminal in the lobula plate.

The dendrite of each digitally reconstructed T4 and T5 was segmented using the brush tool of Amira's segmentation editor, and dendrite volume (Fig. 2A,B) was determined using the material statistics tool of Amira. For comparisons of dendrite volumes across developmental stages, the dendrite volume of each dendrite was normalised to the dendrite volume with the highest value.

To quantify T4 dendrite orientation (Figs 2,5,6; Fig. S1), we imaged dendrites only in frontally oriented regions of the medulla, in which the anteroposterior and dorsoventral axes were recognisable. For each dendrite, we first defined the dendrite's first branching point and made a maximal *z* projection of the whole dendrite in Fiji. Next, we used a custom-written Python script to manually set a threshold in the image to remove background noise, and to calculate a vector from the dendrite's first branching point to every fluorescent pixel. The angles of the calculated vectors were binned in 12 bins, values were normalised to the total number of vectors, and polar histograms were plotted. A similarity index between the dendrite orientation of a *grain*-overexpressing (or a *grain-RNAi* expressing) T4 neuron and the average dendrite orientation of a control T4 subtype (Fig 5S; Fig. 6S) was calculated as follows: the values of equivalent bins in the two polar histograms were subtracted, and all the resulting absolute values were summed. Therefore, the higher the value was (dark blue in Fig 5S; Fig. 6S), the more different the dendrite orientations of the two neurons were. By contrast, the lower the value was (yellow in Fig 5S; Fig. 6S), the more similar the dendrite orientations of the two neurons were. Calculations were performed using Microsoft Excel and Rstudio, and plots were constructed using Rstudio.

### Sample preparation and single cell RNA-sequencing

*Drosophila* pupae of the line *SS00324-Gal4* recombined with *UAS-mCD8::GFP*, were collected at 0 h APF and kept in an incubator at 25°C at 60% humidity. Pupae were put on ice for 15 min before the desired developmental stage and then dissected in Schneider's insect medium (Sigma-Aldrich, S0146) with 10% fetal bovine serum (complete Schneider's medium). The dissociation protocol was modified from a previous study (Harzer et al., 2013). Pupae were dissected for a total of 1 h and washed three times with complete Schneider's medium before an incubation for 30 min at 30°C with a mix of papain (5 units), Liberase TM (0.13 Wu) and complete Schneider's medium in a total volume of 210 µl. Afterwards, they were washed three times with complete Schneider's medium before dissociating the cell bodies by pipetting up and down 15 times with a 200 µl pipette. Next, GFP^+^ cell bodies were isolated from the samples using a BD Aria III cell sorter. Propidium iodine was added as a dead cell marker to remove apoptotic cells. The sorted cells were immediately counted with a haemocytometer and loaded in the 10x Chromium Controller. We aimed to recover between 5000 and 10,000 cells per reaction, dependent on the concentration of the cell suspension. The libraries were prepared as instructed by the 10x Genomics protocol. We used the v. 3 Kit for all reactions. All sequencing runs were performed on an Illumina NextSeq 500 Sequencing System (SY-415-1001) by the next-generation sequencing facility at the Max Planck Institute (MPI) of Biochemistry. The libraries were sequenced with a NextSeq 500/550 High Output Kit v2.5 (75 cycles or 150 cycles, Illumina, 20024906/20024907).

### Single cell RNA-sequencing analysis

The data were preprocessed using the Cell Ranger software v3 (10x Genomics) and aligned to the Ensemble 97 *Drosophila melanogaster* genome. GFP, Gal4 DBD and Gal4 AD (Addgene sequences: #26220, #17574, #26233, #26234) were added to the reference genome and annotation file. The output files of Cell Ranger were loaded into R and analysed with the R package Seurat v3.1.0.9007 (development version). The datasets were manually filtered based on the number of counts per cell and the number of features per cell (Table S1). Genes were only considered if they were expressed in at least three cells and cells with fewer than 200 unique molecular identifiers (UMIs, molecular tags to detect unique mRNA transcripts) were excluded. Furthermore, we removed all cells in which more than 10% of all counts could be allocated to either genes coding for mitochondrial or heatshock proteins (Table S2). These genes are an indicator of a cellular stress response, which can change the transcription profile of affected neurons (Morrow and Tanguay, 2003). The genes were identified by searching the list of detected genes for ‘mt:’ and ‘Hsp’. Gender-specific gene expression can also drive substantial transcriptomic variation that can mask biological signal. To mitigate this effect, we used an approach similar to that proposed in a previous study (Mayer et al., 2018). A gender score for each cell was calculated using a supervised analyses with known gender specific markers (Amrein and Axel, 1997; Mayer et al., 2018). To remove misleading sources of variation, we regressed out the number of UMIs, genes detected per cell, the gender score, as well as the percentages of mitochondrial, heatshock and ribosomal proteins expressed using the SCTransform function in Seurat v3. SCTransform was also used to normalise the expression values. To batch correct the two datasets acquired for each developmental stage, we used the integration tools from Seurat v3. We set the number of variable genes to 10,000 in the SCTransform and the SelectIntegrationFeatures functions. Subsequently, we applied the PrepSCTIntegration and FindIntegrationAnchors functions before combining the datasets with IntegrateData from Seurat v3. The adjusted expression levels were saved in the ‘integrated’ assay of the Seurat object, which was used for the following analysis. After PCA, we used the first 15 principal components (PCs) and a resolution parameter of 0.8 for the clustering of all datasets with the Louvain algorithm. We qualitatively identified and removed clusters that were not T4/T5 neurons or had a different transcriptome because of the cellular stress response, by manually excluding cell clusters that had an unusually high percentage of heatshock and mitochondrial counts (Table S2), as well as clusters with low expression of T4/T5-specific markers (SoxN, Sox102F, Lim1) (Pankova and Borst, 2016; Davie et al., 2018; Konstantinides et al., 2018; Davis et al., 2020). Thus, we were able to discard cells that added noise to the datasets. For the resulting datasets, we first defined the 2000 most variable genes for every developmental stage followed by PCA and clustering, as before, with adjusted parameters (Table S2). The number of PCs used for the clustering was determined manually using the elbow method based on the value of the standard deviation of every PC. We visualised the integrated datasets using uniform manifold approximation and projection (UMAP) and annotated the clusters according to known markers. In order to validate the similarity of clusters between stages, we integrated the datasets from each developmental stage using the CCA alignment tool from Seurat v3. The variable genes were set to 2000 and we used ten PCs for dimensionality reduction and visualization (Fig. 3D).

### Differential gene expression analysis

In order to find differentially expressed genes (DEGs) between T4/T5 subtypes, we performed a pairwise comparison of the annotated clusters using the FindMarkers function of Seurat v3 for all developmental stages separately after the clusters were annotated. We used the ‘RNA’ assay with high thresholds (min.pct=0.5, min.diff.pct=0.5, logfc=2) in order to only find genes that were specific for each cluster. Of the 159 DEGs identified at any of the five developmental stages (Table S3), 16 DEGs passed the thresholds at all stages. For visualization of these genes, we used the ‘integrated’ assay for the heat map (Fig. 3C; Fig. S2). In order to compare the expression of genes, we switched to the ‘RNA’ assay, as it contains the number of UMIs assigned to each gene, without any normalisation (Fig. 4A-G; Fig. S3A). Dot plots were obtained using the DotPlot function of Seurat v3 and the ‘SCT’ assay, which calculated the average expression of each gene in each cluster and represented it by a colour scale. The size of the dots was determined by the percentage of cells expressing the respective gene (Fig. 4H-J, Fig. S3B).

### Identification of transcription factors and cell-membrane proteins in the list of 159 DEGs

In order to identify transcription factors in the list of 159 DEGs, we obtained a list of 651 *Drosophila* transcription factors from the Animal Transcription Factor Database v. 3.0 (bioinfo.life.hust.edu.cn/AnimalTFDB/) (Hu et al., 2019). To identify cell-membrane proteins (excluding neurotransmitter/neuropeptide receptors, ion channels and transporters), we manually inspected the function annotation of each gene in FlyBase (release FB2019_04) (Thurmond et al., 2019). A few genes that were not annotated in FlyBase as cell-membrane proteins were considered as cell-membrane proteins based on previous work (Li et al., 2017).

## Supplementary Material

Supplementary information

Reviewer comments
