# Supplementary material for: A combinatorial code of transcription factors specifies subtypes of visual motion-sensing neurons in Drosophila
Source: Development. 2020 May 13;147(9):dev186296. doi: 10.1242/dev.186296 (PMC7240302; doi:10.1242/dev.186296)
Supplement: Supplementary information [file develop-147-186296-s1.pdf]

Figure S1

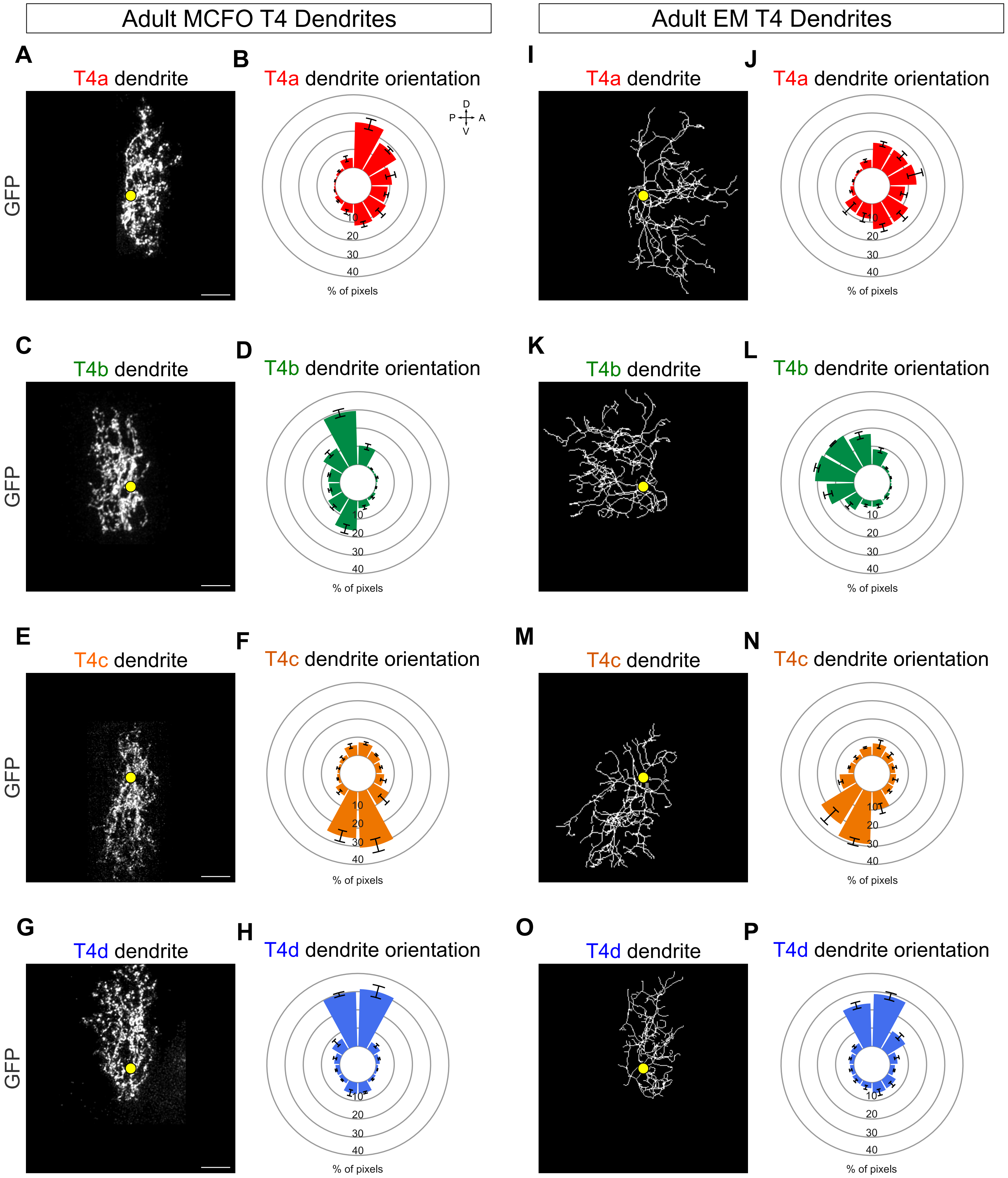

Development • Supplementary information

Figure S1. Quantification of dendrite orientation in the four T4 subtypes imaged by confocal microscopy or reconstructed from electron microscopy data.

(A-H) Examples of adult T4a, T4b, T4c and T4d dendrites imaged with confocal microscopy after labelling by means of the MCFO and the *SS00324-splitGal4.* Yellow dots mark the dendrite´s first branching point. Scale bars: 5 µm. Quantifications of dendrite orientation are shown as polar histograms with the 2D distribution of fluorescent pixels (indicative of the presence of dendritic branches) around the dendrite´s first branching point (N=4 dendrites per subtype). A, P, D, V: Anterior, Posterior, Dorsal, Ventral (visual field coordinates). Mean ± SEM are shown.

(I-P) Examples of adult T4a, T4b, T4c and T4d dendrites reconstructed from electron microscopy (EM) data (Takemura et al., 2017). Yellow dots mark the dendrite´s first branching point. Quantifications of dendrite orientation are shown as in (A-H) (N=4 dendrites per subtype).

Figure S2

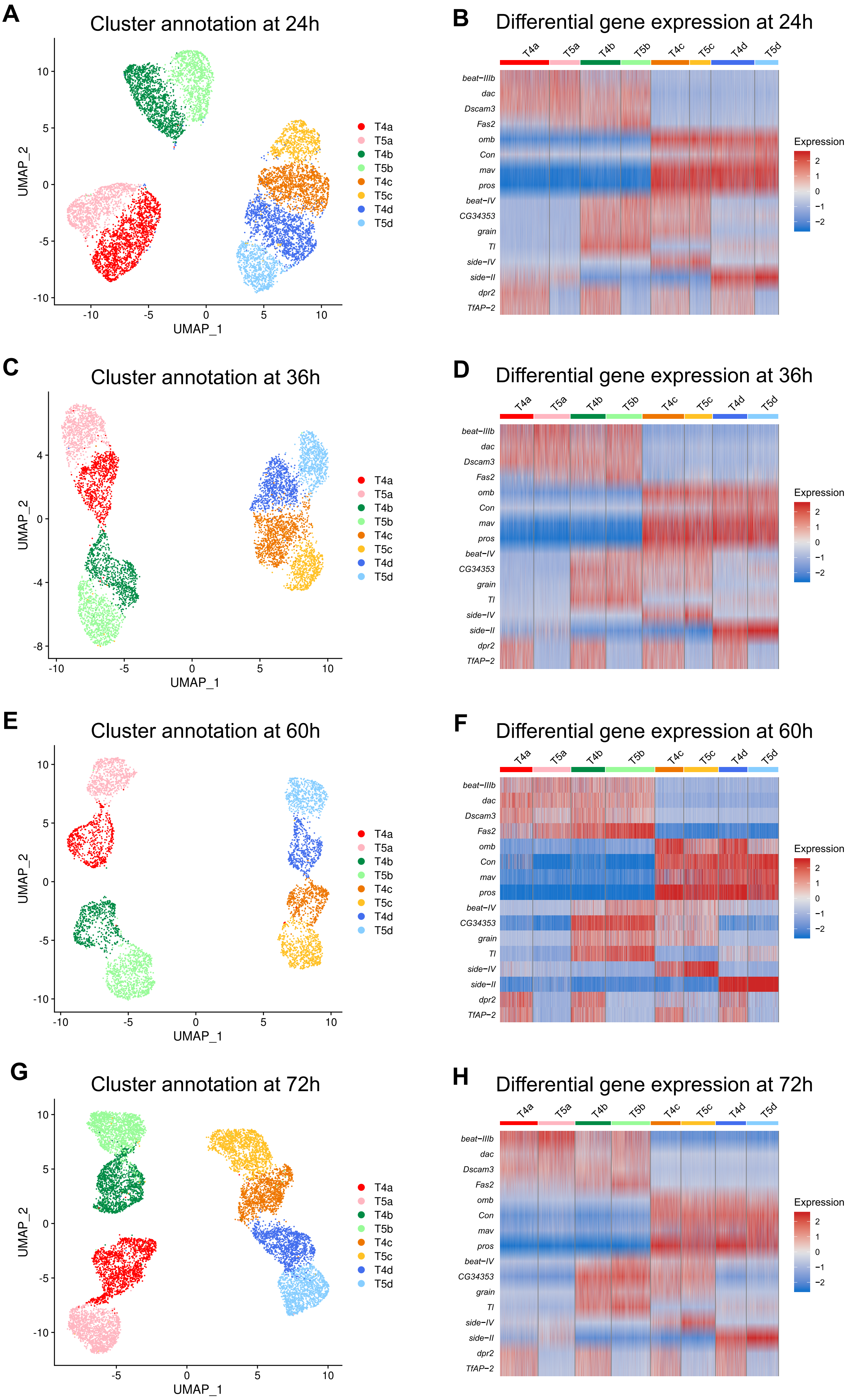

Development • Supplementary information

Figure S2. Eight transcriptionally distinct groups of T4/T5 neurons correspond to the four subtypes of T4 and T5 neurons at 24, 36, 60 and 72h APF.

(A-H) Visualizations of T4/T5 neurons sequenced either at 24, 36, 60 or 72h APF (A,C,E,G) using UMAP after dimensionality reduction by PCA and unsupervised clustering based on the Louvain algorithm. Each dot is a single cell. Cells are arranged according to transcriptome similarity. We manually assigned clusters to either T4 or T5 based on *TfAP-2* expression. Clusters were assigned to either T4/T5a,b or T4/T5c,d based on *dac* and *omb* expression. We assigned clusters to either T4/T5b,c or T4/T5a,d based on *grain* expression. Eight single-cell clusters were matched to the four T4 subtypes (T4a-d) and to the four T5 subtypes (T5a-d) in every examined developmental stage. Heat maps (B,D,F,H) show the expression levels of the 16 genes found to be differentially expressed between the single-cell clusters of T4 and T5 subtypes in every developmental stage examined. Each column corresponds to a cell and each row corresponds to a gene. Cells are grouped based on cluster identities. Genes were manually ordered based on visual inspection of subtype-specific expression patterns.

Development • Supplementary information

Figure S3

A

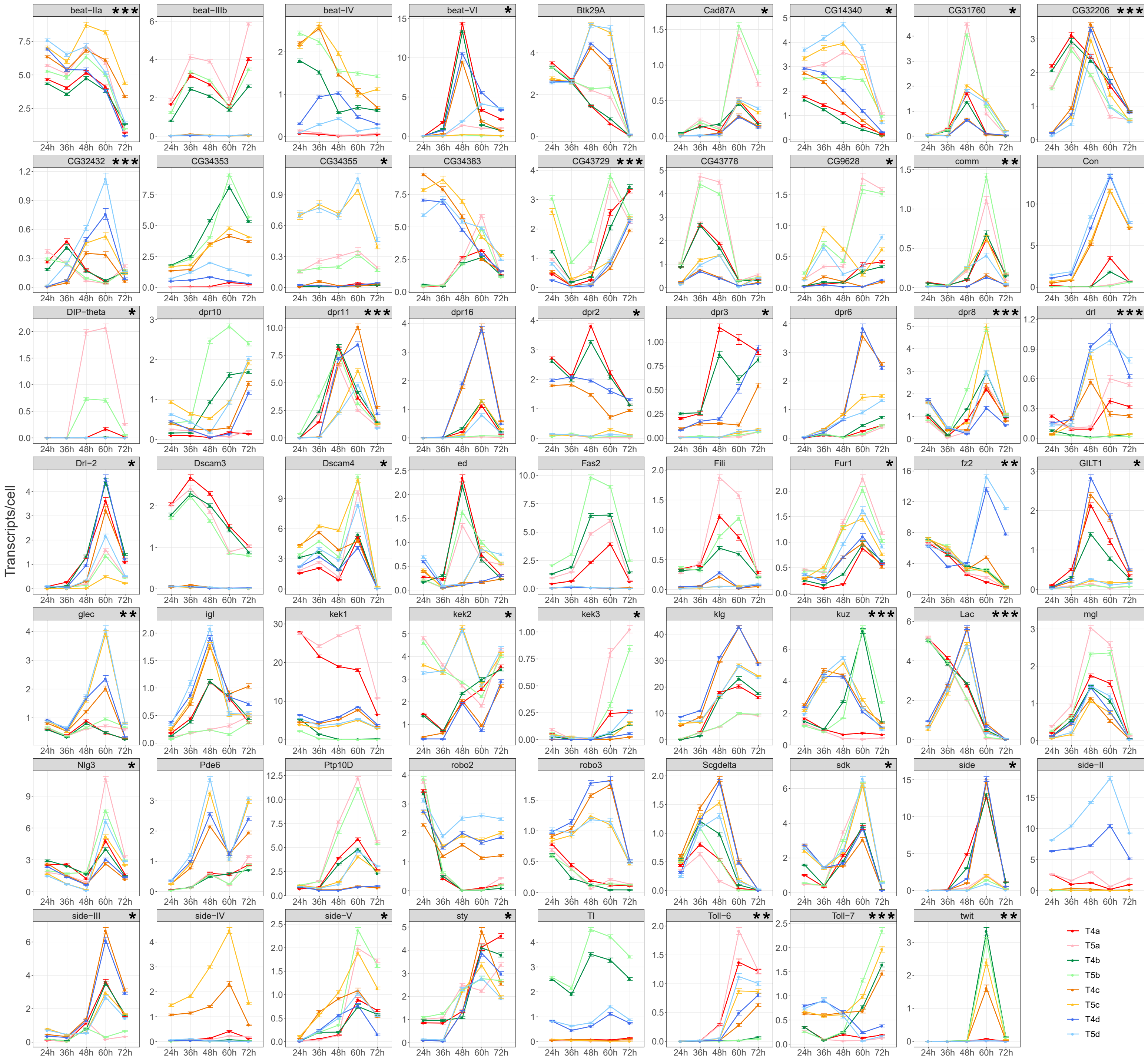

B

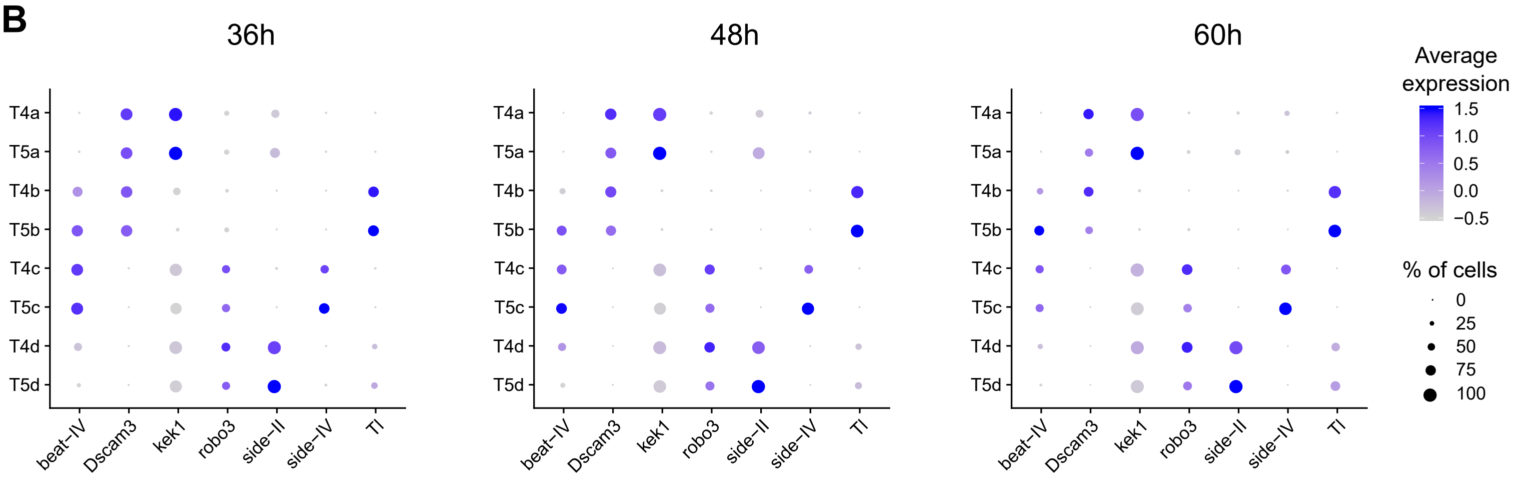

Development • Supplementary information

Figure S3. Expression patterns of cell-membrane proteins differentially expressed between T4/T5 subtypes.

(A) Subtype-specific expression patterns and dynamics of all genes encoding for cell-membrane proteins that were found differentially expressed between T4/T5 subtypes. Y axis shows the count of transcripts per cell (mean ± SEM). X axis shows developmental stage (h APF). Genes were arranged alphabetically. *: Genes with either higher expression levels in all T4 than in all T5 subtypes, or vice versa, or with subtype-specific expression patterns only in T4 or T5 neurons. **: Genes differentially expressed between T4/T5 subtypes only during the last phase of dendrite growth (60-72h APF). ***: Genes with subtype-specific expression patterns that switch over time.

(B) Dot plots showing the mean scaled expression levels (colour-coded) of some cell-membrane proteins in the different T4/T5 subtypes at 36, 48 and 60h APF. Dot sizes represent the percentage of cells in which the gene was detected. Genes were arranged alphabetically.

Figure S4

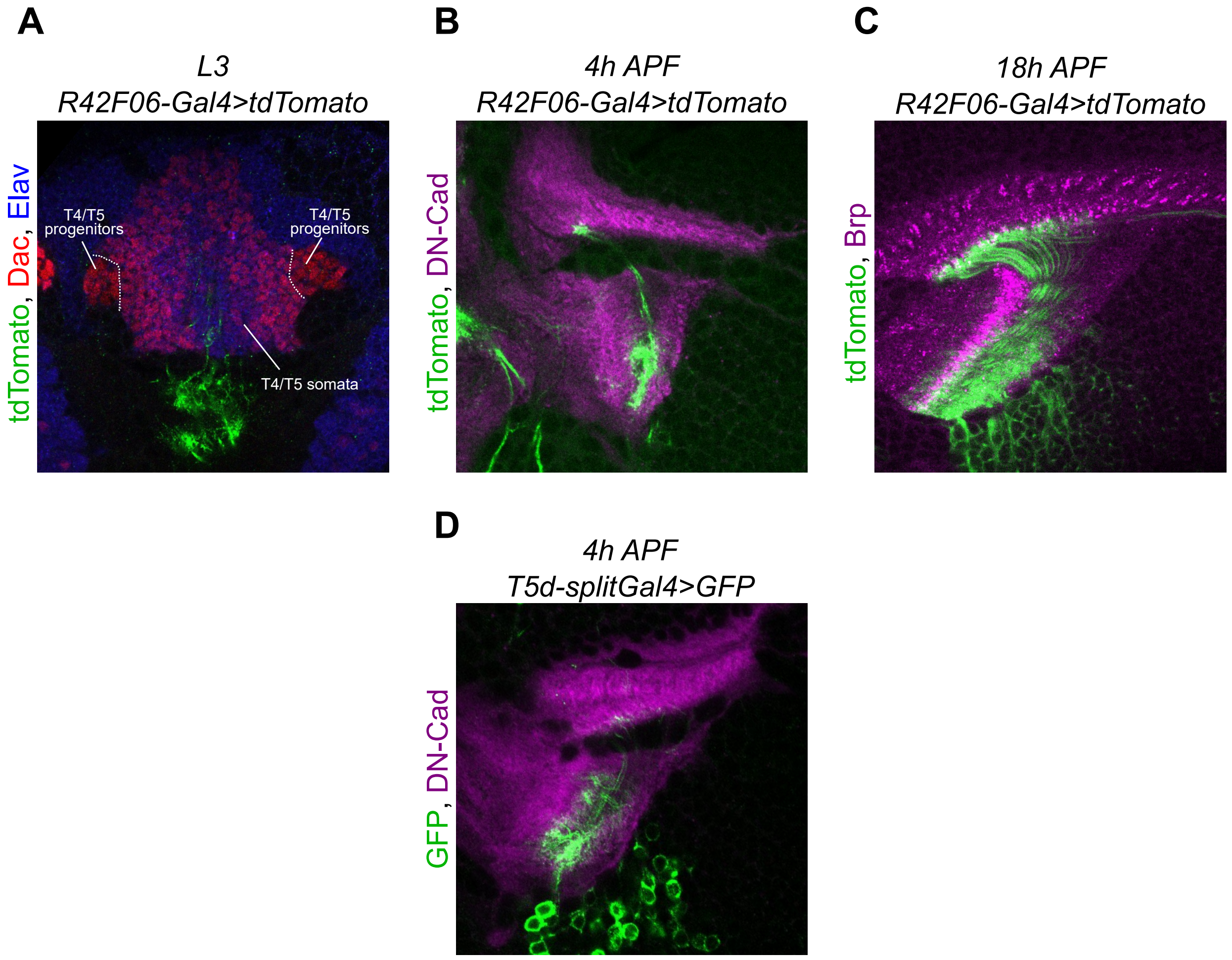
A
L3
R42F06-Gal4>tdTomato
tdTomato, Dac, Elav
T4/T5 progenitors
T4/T5 progenitors
T4/T5 somata
B
4h APF
R42F06-Gal4>tdTomato
tdTomato, DN-Cad
C
18h APF
R42F06-Gal4>tdTomato
tdTomato, Brp
D
4h APF
T5d-splitGal4>GFP
GFP, DN-Cad

Figure S4. Characterization of *enhancer-Gal4* driver lines used for *grain* overexpression in T4/T5 neurons.

(A) The *R42F06-Gal4* line labels only a very few maturing T4/T5 neurons in the late L3 larval optic lobe. Neuronal cell bodies were marked with anti-Elav. T4/T5 neuron progenitors (Dac+/Elav-) are not labelled by the *R42F06-Gal4* line.

(B,C) T4/T5 neurons expressing tdTomato under the control of *R42F06-Gal4* in pupal optic lobes at 4h and 18h APF. The *R42F06-Gal4* expression pattern follows the maturation wave of T4/T5 neurons (Pinto-Teixeira et al., 2018). Anti-DN-Cadherin (DN-Cad) and anti-Bruchpilot (Brp) label the neuropils.

(D) Early pupal optic lobe (4h APF) showing T5 neurons expressing GFP by means of the *T5d-splitGal4* driver line.

Development • Supplementary information

Figure S5

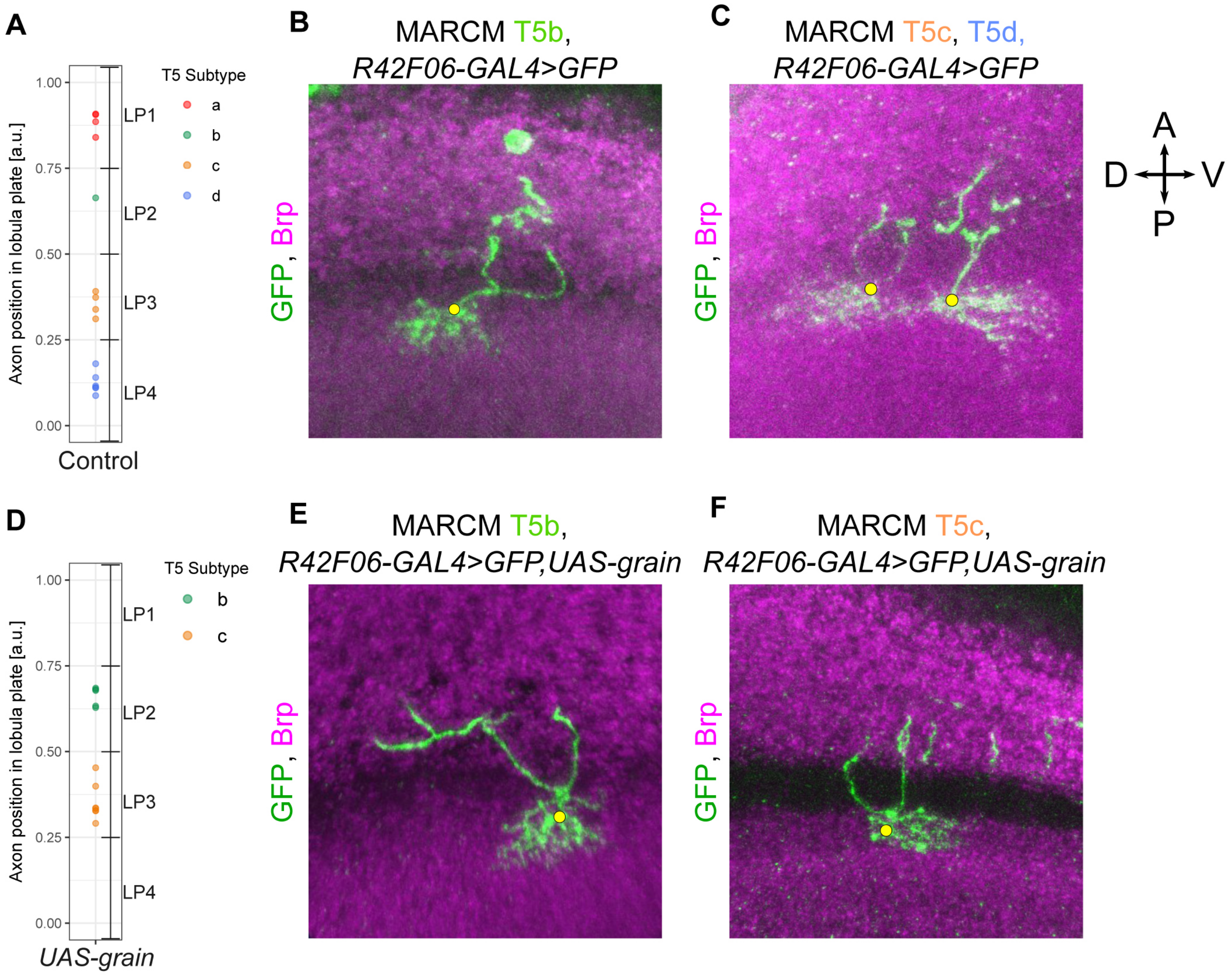
A
1.00
0.75
0.50
0.25
0.00
Axon position in lobula plate [a.u.]
LP1
LP2
LP3
LP4
Control
T5 Subtype
a
b
c
d
B
MARCM T5b,
R42F06-GAL4>GFP
GFP, Brp
C
MARCM T5c, T5d,
R42F06-GAL4>GFP
GFP, Brp
A
D
V
P
D
1.00
0.75
0.50
0.25
0.00
Axon position in lobula plate [a.u.]
LP1
LP2
LP3
LP4
UAS-grain
T5 Subtype
b
c
E
MARCM T5b,
R42F06-GAL4>GFP,UAS-grain
GFP, Brp
F
MARCM T5c,
R42F06-GAL4>GFP,UAS-grain
GFP, Brp

Figure S5. *Grain* overexpression in developing T5 neurons results in adult optic lobes with only T5b,c neurons.

(A) Positions in the lobula plate occupied by axon terminals of single, control T5 neurons labelled by MARCM (N=15). Each T5 neuron was classified into one of the four subtypes based on the lobula plate layer occupied by its axon (T5a: N=4, T5b: N=1, T5c: N=4, T5d: N=6).

(B) 3D visualization of the dendrite from a control T5b neuron (axon in lobula plate layer 2) labelled by MARCM. Yellow dot marks the dendrite´s first branching point. Anti-Bruchpilot (Brp) labels the neuropils. A, P, D, V: Anterior, Posterior, Dorsal, Ventral (visual field coordinates).

(C) 3D visualization of the dendrites from a control T5d (left, axon in lobula plate layer 4) and a control T5c (right, axon in lobula plate layer 3) labelled by MARCM.

(D) Positions in the lobula plate occupied by axon terminals of single, *grain*-overexpressing T5 neurons labelled by MARCM (N=12). *Grain*-overexpressing T5 neurons project axons to either lobula plate layer 2 (N=5) or lobula plate layer 3 (N=7).

(E,F) 3D visualizations of the dendrites from grain-overexpressing T5b (axon in lobula plate layer 2) and T5c (axon in lobula plate layer 3) labelled by MARCM. The dendrite orientations of these neurons are indistinguishable from those of T5b and T5c wild-type neurons (B,C). The orientation of T5 dendrites was qualitatively assessed by visual inspection.

Development • Supplementary information

Figure S6

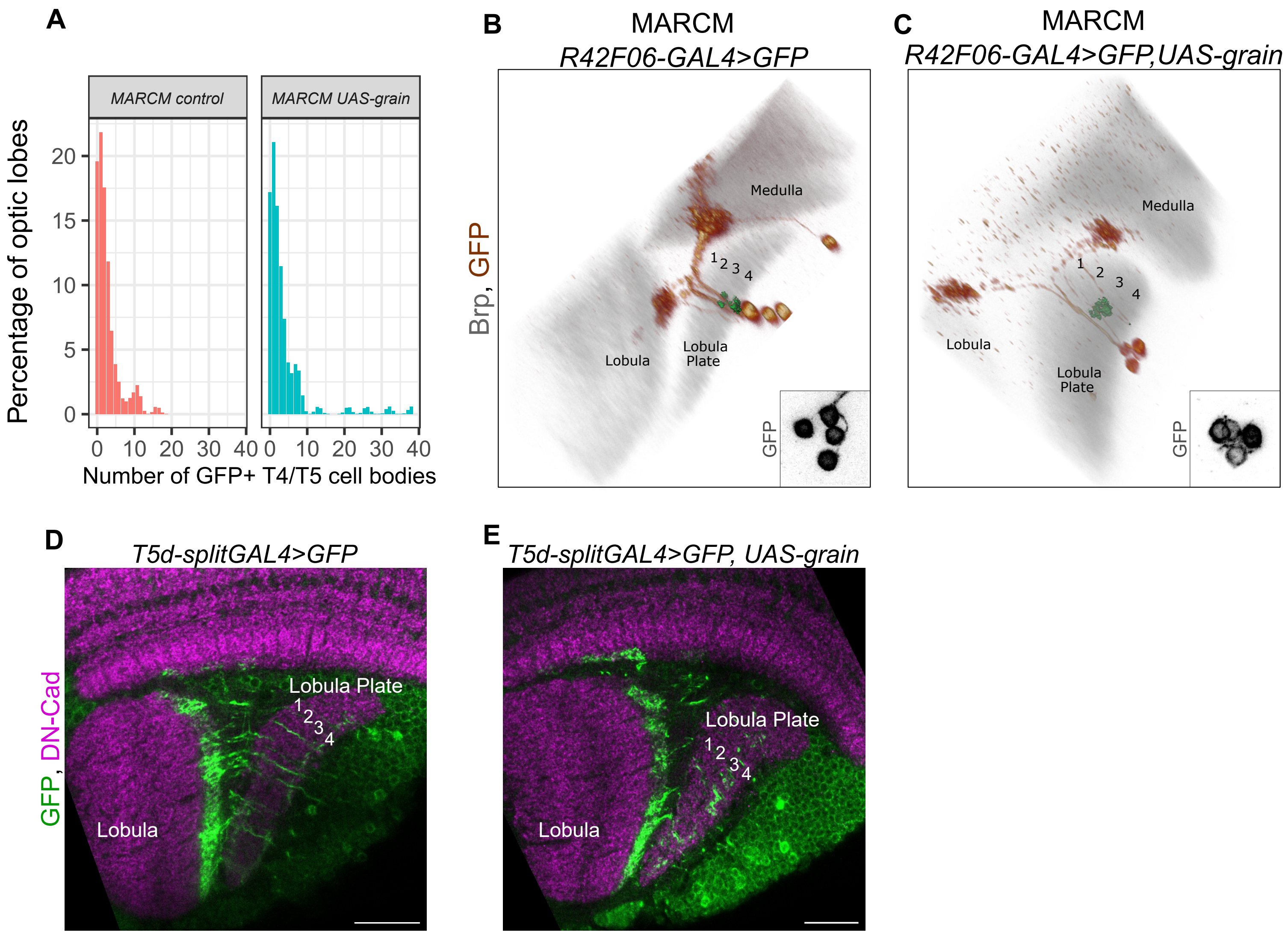

Development • Supplementary information

Figure S6. ***Grain*** **overexpression in developing T4/T5 neurons does not cause specific cell death of T4/T5a,d subtypes.**

(A) Histograms showing the percentages of optic lobes (Y Axis) found with different numbers of GFP^+ T4 and T5 cell bodies (X Axis) in control MARCM experiments and in *grain* overexpression MARCM experiments.

(B) 3D visualization of two T4 and two T5 neurons labelled in a control MARCM experiment, and projecting dendrites and axons to the same retinotopic position of the medulla, lobula and lobula plate. The inset shows the cell bodies of these neurons. The axon terminals of the four neurons (in green, digitally reconstructed) were located in layers 3 and 4 of the lobula plate. These T4c,T5c,T4d,T5d neurons represent a four-cell clone produced by a single neuroblast (Pinto-Teixeira et al., 2018).

(C) 3D visualization of two T4 and two T5 neurons labelled in a *grain* overexpression MARCM experiment, and projecting dendrites and axons to the same retinotopic position of the medulla, lobula and lobula plate. Their cell bodies are shown in the inset. The axon terminals of the four neurons (in green, digitally reconstructed) were located only in layer 3 of the lobula plate. These T4c,T5c,T4c,T5c neurons represent a four-cell clone produced by a single neuroblast.

(D) The *T5d-splitGal4* line labels mainly T5 neurons with axons in layer 4 of the lobula plate (T5d subtype) at the adult stage. Anti-DN-Cadherin (DN-Cad) labels the neuropils.

(E) T5 neurons are still present at the adult stage upon overexpression of *grain* with the *T5d-splitGal4* line. T5 neurons have axons in layer 3 of the lobula plate in this condition, consistent with T5d transforming into T5c subtype after gaining *grain* expression.

Development • Supplementary information

Table S1. Output information from the cellranger pipeline and additional information concerning the filtering for every dataset.

Click here to Download Table S1

Table S2. Parameters used for different steps of the scRNA-seq analysis.

Click here to Download Table S2

Development • Supplementary information

Mitochondrial Genes:

mt:ND2, mt:tRNA:Trp-TCA, mt:tRNA:Tyr-GTA, mt:CoI, mt:CoII, mt:ATPase8, mt:ATPase6, mt:CoIII, mt:tRNA:Gly-TCC, mt:ND3, mt:tRNA:Ala-TGC, mt:ND5, mt:ND4, mt:ND4, mt:ND6, mt:Cyt-b, mt:ND1, mt:tRNA:Leu-TAG, mt:lrRNA, mt:srRNA, mt:ori, mt:tRNA:Ile-GAT, mt:tRNA:Cys-GCA, mt:tRNA:Lys-CTT, mt:tRNA:Arg-TCG, mt:tRNA:Ser-TGA, mt:tRNA:Val-TAC

Heat Shock Proteins:

Hsp70Aa, Hsp70Ab, Hsp70Ba, Hsp70Bbb, Hsp70Bb, Hsp70Bc, Hsp68, Hsp83, Hsp67Bc, Hsp26, Hsp67Ba, Hsp23, Hsp27, Hsp60A, Hsp60D

Male-specific Genes:

lncRNA:roX1, lncRNA:roX2

Differentially Expressed Genes:

5-HT1A, AANAT1, ab, Adk1, ana, app, AstC-R2, beat-IIa, beat-IIIb, beat-IV, beat-VI, bi, bnb, Btk29A, Ca-alpha1T, Cad87A, Ccn, CG10384, CG11191, CG11319, CG12643, CG13739, CG14340, CG15236, CG15765, CG1688, CG17124, CG17716, CG17839, CG2016, CG2082, CG2269, CG30015, CG31221, CG31324, CG31637, CG31676, CG31690, CG31760, CG32204, CG32206, CG32333, CG32432, CG33143, CG33543, CG33639, CG34347, CG34353, CG34355, CG34377, CG34383, CG3655, CG42339, CG42541, CG42817, CG4341, CG43427, CG43729, CG43778, CG43902, CG45263, CG4546, CG6006, CG6959, CG7991, CG8861, CG9331, CG9628, CG9932, cmpy, comm, Con, dac, DIP-theta, dpr10, dpr11, dpr16, dpr2, dpr3, dpr6, dpr8, drl, Drl-2, Dscam3, Dscam4, ed, Fas2, Fili, fred, Frq1, Fur1, fz2, Gadd45, GILT1, glec, Grd, grn, hig, Hs3st-A, igl, jus, kek1, kek2, kek3, klg, kuz, Lac, lncRNA:CR44978, mAChR-B, mav, mgl, mspo, Nlg3, nolo, Nost, NPFR, Oatp26F, Octalpha2R, Octbeta2R, osp, Pde1c, Pde6, Pgant2, pHCl-1, pros, Ptp10D, PVRAP, px, rad, RapGAP1, Rgk2, robo2, robo3, sano, Scgdelta, sdk, Sf3b6, Shawl, side, side-II, side-III, side-IV, side-V, SKIP, SLO2, Slob, SPR, sty, Svil, Tet, TfAP-2, Tl, Toll-6, Toll-7, Trim9, TrissinR, twit, twz, zld

Table S3. Genes used for filtering the datasets and genes identified during the differential gene expression analysis.

Development • Supplementary information
