# Supplementary material for: A combinatorial code of transcription factors specifies subtypes of visual motion-sensing neurons in Drosophila
Source: Development. 2020 May 13;147(9):dev186296. doi: 10.1242/dev.186296 (PMC7240302; doi:10.1242/dev.186296)
Supplement: Reviewer comments [file develop-147-186296_review_history.pdf]

A combinatorial code of transcription factors specifies subtypes of visual motion-sensing neurons in *Drosophila*

Nikolai Hörmann, Tabea Schilling, Aicha Haji Ali, Etienne Serbe, Christian Mayer, Alexander Borst and Jesús Pujol-Martí
DOI: 10.1242/dev.186296

Editor: Francois Guillemot

Review timeline

Original submission:	11 November 2019
Editorial decision:	11 December 2019
First revision received:	19 February 2020
Editorial decision:	11 March 2020
Second revision received:	12 March 2020
Accepted:	20 March 2020

Original submission

First decision letter

MS ID#: DEVELOP/2019/186296

MS TITLE: A combinatorial code of transcription factors specifies subtypes of visual motion-sensing neurons in Drosophila

AUTHORS: Nikolai Hörmann, Tabea Schilling, Aicha Haji Ali, Christian Mayer, Alexander Borst, and Jesús Pujol-Martí

I have now received the reports of three referees on your manuscript and I have reached a decision. The referees' comments are appended below, or you can access them online: please go to BenchPress and click on the 'Manuscripts with Decisions' queue in the Author Area.

As you will see, all the referees express great interest in your work, but referees 2 and 3 also have significant criticisms and recommend a substantial revision of your manuscript before we can consider publication. If you are able to revise the manuscript along the lines suggested, which may involve further experiments, I will be happy to receive a revised version of the manuscript.. Your revised paper will be re-reviewed by the original referees, and its acceptance will depend on your addressing satisfactorily all their major concerns. Please also note that Development will normally permit only one round of major revision.

Please attend to all of the reviewers' comments and ensure that you clearly highlight all changes made in the revised manuscript. Please avoid using 'Tracked changes' in Word files as these are lost in PDF conversion. I should be grateful if you would also provide a point-by-point response detailing how you have dealt with the points raised by the reviewers in the 'Response to Reviewers' box. If you do not agree with any of their criticisms or suggestions please explain clearly why this is so.

© 2020. Published by The Company of Biologists under the terms of the Creative Commons Attribution License (https://creativecommons.org/licenses/by/4.0/).

Reviewer 1

Advance summary and potential significance to field

Review of "A combinatorial code of transcription factors specifies subtypes of visual motion sensing neurons in Drosophila" by Hoermann/Pujol-Marti.

This is a interesting and excellent paper using single cell RNAseq to identify gene expression profiles of TFs and CSMs unique for the subtypes of T4/T5 neurons in the visual system of Drosophila. It is essentially the same as a very recent paper by the Zipursky lab (Kurmangaliyev et al. eLife 2019;8:e50822. DOI: https://doi.org/10.7554/eLife.50822) but that is no reason for not publishing this manuscript. Indeed, both papers taken together provide a better view of the system than either paper alone.

Comments for the author

I have no major comments. This is a very nice paper.

Minor comments (to be addressed if editor or author agrees).

1. The last sentence of the abstract states "... a combinatorial code of transcription factors (is) ... required in T4/T5 neurons for detecting motion..." I don't see any behavioral data that supports this conclusion. Perhaps the long concluding sentence can be broken into two parts and this functional conclusion left out.

2. The last sentence of the introduction states "... our results reveal a combinatorial code of transcription factors diversifying the mnorphologies of T4/T5 neuron subtypes..." Again I only see data on Grain regulation of neuronal morphology – the other combinatorial codes are just hypothesized to regulate neuronal morphology by analogy to Grain. I think this conclusion sentence should be toned down to leave space for future studies to perform novel functional tests of the other TF combinations.

3. Figure 1B and 1C panels are too small to be useful. Please delete (they seem optional to me) or find a way to present more clearly.

4. line 141 the Davis pre-print should be a personal communication or a citation to a BioRxiv paper perhaps? And with a year of 2018 it should be out by now?

5. Please mention when the T4/T5 gal4 lines are first expressed. Are they in progenitors or just post-mitotic neurons? If the latter, are they expressed before or after neuronal axon/dendrite target selection?

6. line 399 please give more detail on how Amira was used to quantify dendrite volume. Is there a specific function in Amira that you can name? And how boundary thresholds were established?

Reviewer 2

Advance summary and potential significance to field

This study identifies the Grain transcription factor that distinguishes the b/c from a/d subtypes of T4 and T5 motion detection neurons in the Drosophila visual system. Ectopic Grain potently transformed the a/d to b/c subtypes based on dendrite orientations as well as axon projection patterns, elegantly shown with MARCM.
This finding, together with other known TFs that diversify the a/b versus c/d subtypes, led to the conclusion that these four direction-selective subtypes of T4/T5 neurons in the Drosophila visual system are defined by simple combinatorial TF codes.

© 2020. Published by The Company of Biologists under the terms of the Creative Commons Attribution License (https://creativecommons.org/licenses/by/4.0/).

Comments for the author

However, this study did not address loss-of-Grain phenotypes, which can be carried out with twin-spot MARCM, to knock out grain selectively from one of the two last-born GMCs. Given the above model removing Grain from the Notch[off] GMC (as judged from its paired sister clone) should transform b/c to a/d.
Alternatively, one can confirm production of four Grain+ neurons by a Notch mutant NB, and examine if repressing Grain (e.g. by RNAi) could convert the four b or c neurons (as reported by Filipe Pinto-Teixeira et al., 2018) to four a or d neurons in the four-cell Notch mutant clones.

Reviewer 3

Advance summary and potential significance to field

The main question of this study is “what are the molecular basis of neuronal cell-type specification?” As a model, the authors used T4/T5 neurons of Drosophila visual system, which are well described anatomically and functionally. These cells derive from the same progenitor pool, although during development they form eight distinct cell-types with unique innervation position in Lobula plate and dendritic arbor direction.
These morphological features also determine the specific function of each cell-type in fly motion detection vision. In this work Hoermann et. al. wanted to understand how these unique features could be acquired.
To answer the main question of the study, the authors first determined a temporal window in which dendritic branches are formed. Subsequently they performed scRNA-seq and compared transcriptomes of T4/T5 cell-types in different developmental stages. After detailed analysis they found a combinatorial regulatory code of five transcriptional factors that regulates development of T4/T5 dendrite orientation and axon projection patterns.
In my opinion the strong part of the paper is an accurate analysis of transcriptional profiles from scRNA-seq data, in which they found a range of differentially expressed genes within eight T4/T5 clusters in different time points, which could play variety of roles in cells development.
Additionally, it was clearly shown that a “minimal” transcription factor code of five TFs could reflect identity of all eight cell types.
The phenotype observed upon Grain missexpression is a strong indicator that manipulating this code changes cell-type identity.
In general, I find this work original, interesting and well done. The work is well illustrated, the figures are structured and easy to interpret.

Comments for the author

To further consolidate their conclusions, I have a few recommendations:
1) For Grain levels quantifications the authors used Grain-Gal4 combined with UAS-CD8::GFP. Concluding quantitative expression dynamics of a transcription factor (or any protein for that matter) from a Gal4 reporter construct is highly unreliable. I would suggest the authors use either an endogenous tagged version of the Grain protein instead (if available) or at least a UAS-Grain tagged with a small tag such as V5. At minimum the authors should be extremely cautious about making strong conclusions from these experiments.
2) An important aspect missing from this study, is whether manipulation of the TF code is sufficient to alter circuit function. To this end, the authors should test whether overexpression of Grain in all T4/T5 changes the optomotor response in the direction predicted by cell fate transformation. (Did these changes impaired postsynaptic connectivity of T4/T5 and could all of the b and c cells find the corresponding partners?)
3) The authors suggest that selective expression of Grain in T4/T5 b and c subtypes is regulated by Notch signaling. The authors should test if Notch activation or inhibition in T4/T5 is sufficient to alter Grain. They could the use T4/T5 LexA driver (Strother 2017) combined with a LexAop-Notch-intra (Weinberger 2017) and examine the cell type expression of Grain-Gal4>UAS-GFP.

© 2020. Published by The Company of Biologists under the terms of the Creative Commons Attribution License (https://creativecommons.org/licenses/by/4.0/).

First revision

Author response to reviewers' comments

Response to reviewers

We are thankful to the three reviewers for their positive comments and insightful suggestions. We provide a point-by-point response to the comments of the reviewers in the following section. In order to incorporate the new results into the revised manuscript, we have included one new main figure (**Fig. 6**) and one new supplemental figure (**Fig. S4**). We have also added **Fig. S6D,E** panels to further support the conclusions of this figure, and we have replaced **Fig. 1B,C** and **Fig. 1F-I** by new panels that are more obvious and did not change the conclusions of the figure. Finally, we have reorganized the data in **Fig. 5S** panel and changed its colour code to show it more clearly. All changes in the text have been highlighted in the new version of the manuscript.

Reviewer 1 Comments for the Author...

I have no major comments. This is a very nice paper.

Minor comments (to be addressed if editor or author agrees).

1. The last sentence of the abstract states "... a combinatorial code of transcription factors (is) ... required in T4/T5 neurons for detecting motion..." I don't see any behavioral data that supports this conclusion. Perhaps the long concluding sentence can be broken into two parts and this functional conclusion left out.

We have changed this concluding sentence to avoid giving the impression that this study directly demonstrates a link between morphology and function, as suggested by the reviewer.

2. The last sentence of the introduction states "... our results reveal a combinatorial code of transcription factors diversifying the mnorphologies of T4/T5 neuron subtypes..." Again I only see data on Grain regulation of neuronal morphology - the other combinatorial codes are just hypothesized to regulate neuronal morphology by analogy to Grain. I think this conclusion sentence should be toned down to leave space for future studies to perform novel functional tests of the other TF combinations.

We thank the reviewer for this suggestion. It was not our intention to make any overstatement, and we agree that this last introduction sentence contained some points that are better suited for the discussion. Therefore, we now finish the introduction with a sentence stating the clearest conclusion from our work, and we have a paragraph in the discussion about the possible roles of the other transcription factors not tested in this study, emphasizing that future studies will need to investigate their roles.

3. Figure 1B and 1C panels are too small to be useful. Please delete (they seem optional to me) or find a way to present more clearly.

We have changed **Fig. 1B,C** panels to present the data more clearly.

4. line 141 the Davis pre-print should be a personal communication or a citation to a BioRxiv paper perhaps? And with a year of 2018 it should be out by now?

During the revision of our manuscript, the Davis pre-print has been published in *eLife*. We have changed this reference accordingly.

5. Please mention when the T4/T5 gal4 lines are first expressed. Are they in progenitors or just post-mitotic neurons? If the latter, are they expressed before or after neuronal axon/dendrite target selection?

© 2020. Published by The Company of Biologists under the terms of the Creative Commons Attribution License (https://creativecommons.org/licenses/by/4.0/).

We have incorporated this information and supporting data (**Fig. S4**) in the new version of the manuscript.

6. line 399 please give more detail on how Amira was used to quantify dendrite volume. Is there a specific function in Amira that you can name? And how boundary thresholds were established?

We have incorporated all this information in the new version of the manuscript.

Reviewer 2 Comments for the Author...

However, this study did not address loss-of-Grain phenotypes, which can be carried out with twin-spot MARCM, to knock out grain selectively from one of the two last-born GMCs. Given the above model, removing Grain from the Notch[off] GMC (as judged from its paired sister clone) should transform b/c to a/d. Alternatively, one can confirm production of four Grain+ neurons by a Notch mutant NB, and examine if repressing Grain (e.g. by RNAi) could convert the four b or c neurons (as reported by Filipe Pinto-Teixeira et al., 2018) to four a or d neurons in the four-cell Notch mutant clones.

We agree that *grain* loss of function phenotypes are desired to further support our model, which predicts that removing *grain* should transform T4/T5b and T4/T5c into T4/T5a and T4/T5d, respectively, as pointed out by the reviewer. To test this, we have done MARCM experiments to express *grain-RNAi* in individual, developing T4 neurons of all subtypes (**Fig. 6**). In this condition, all but one of the single-labelled, adult T4 neurons have axons in either lobula plate layer 1 or 4, which are normally innervated by T4/T5a or T4/T5d subtypes, respectively. T4 neurons expressing *grain-RNAi* that innervated either lobula plate layer 1 or 4 showed corresponding dendrite orientations of T4/T5a or T4/T5d subtypes. These data further support that *grain* differentiates T4/T5b from T4/T5a and T4/T5c from T4/T5d morphologies.

The reviewers will notice that we have used different *Gal4* driver lines for *grain* overexpression MARCM (*R42F06-Gal4*) and for *grain-RNAi* MARCM (*R39H12-Gal4*) experiments. The reason is that expressing *grain-RNAi* with the *R42F06-Gal4* did not affect the axon projection patterns of T4/T5 neurons (**Fig. R1**), while expressing *grain-RNAi* with the *R39H12-Gal4* produced changes in T4/T5 axon projection patterns (**Fig. 6A,B**). This difference most likely results from differences in the expression levels and temporal patterns of the two *Gal4* lines: while the *R39H12-Gal4* shows very strong expression in all T4/T5 neurons from late L3 larval stage onwards (see Fig. S1B from Schilling et al., 2019), expression driven by the *R42F06-Gal4* starts later and seems to be weaker (**Fig. S4A-C**).

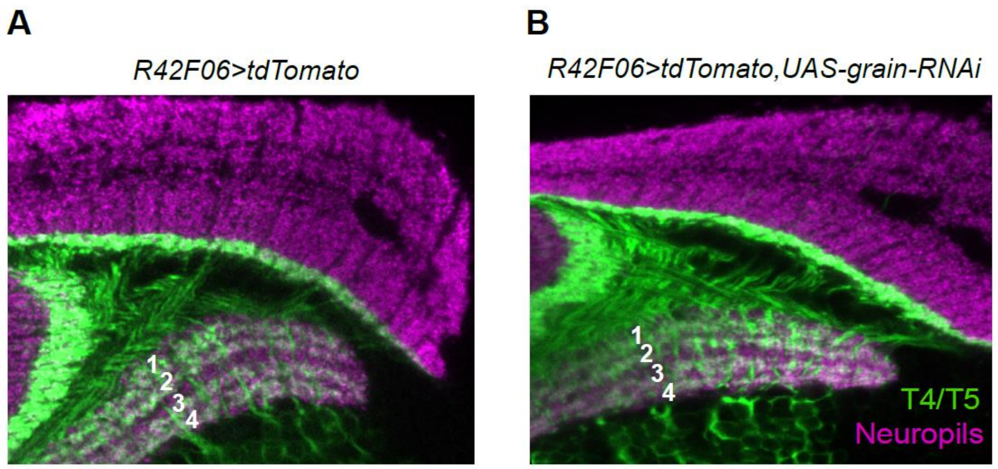

Figure R1. Horizontal views of adult optic lobes showing wild-type T4/T5 neurons (A), and T4/T5 neurons expressing *grain-RNAi* with the *R42F06-Gal4* driver line (B). In both cases, T4/T5 axons form four layers within the lobula plate. Anti-Bruchpilot was used to label the neuropils.

© 2020. Published by The Company of Biologists under the terms of the Creative Commons Attribution License (https://creativecommons.org/licenses/by/4.0/).

Reviewer 3 Comments for the Author...
To further consolidate their conclusions, I have a few recommendations:

1) For Grain levels quantifications the authors used Grain-Gal4 combined with UAS-CD8::GFP. Concluding quantitative expression dynamics of a transcription factor (or any protein for that matter) from a Gal4 reporter construct is highly unreliable. I would suggest the authors use either an endogenous tagged version of the Grain protein instead (if available) or at least a UAS-Grain tagged with a small tag such as V5. At minimum the authors should be extremely cautious about making strong conclusions from these experiments.

We agree that concluding quantitative expression dynamics of a protein from a *Gal4* driver line is unreliable, and we would like to emphasize that we did not use the *grain-Gal4* driver line for this purpose. The *grain-Gal4* was used in combination with two other transgenic lines, scRNA-seq data, and anti-Grain immunostaining to conclude that *grain* is specifically expressed in T4/T5b,c subtypes during development. Our scRNA-seq analysis revealed that *beat-IV*, *CG34353* and *grain* are co-expressed in the same single-cell clusters at all examined developmental stages (**Fig. 3C**, **Fig. S2**). The examination of the *beat-IV-GFP* and *CG34353-GFP* MIMIC lines (endogenous GFP-tagged version of the proteins), as well as of the *grain-Gal4* line combined with *UAS-RFP*, showed fluorescent protein expression specifically in T4/T5b,c neurons at 48h APF (**Fig. 3E-G**). Next, we performed anti-Grain immunostaining and found higher anti-Grain signal in *grain-Gal4*$^+$ T4/T5 cell bodies than in *grain-Gal4*$^-$ T4/T5 cell bodies (**Fig. 3H,I**). Remarkably, no *grain* transcripts were detected in T4/T5a,d cells in our scRNA-seq quantitative analysis along five different developmental stages, while *grain* transcripts were detected in T4/T5b,c cells at all these stages (**Fig. 4D**). Based on all these results, we are confident that *grain* is specifically expressed in T4/T5b,c subtypes. In order to explain these results more clearly, we have modified **Fig. 3E-I** legends and captions.

2) An important aspect missing from this study, is whether manipulation of the TF code is sufficient to alter circuit function. To this end, the authors should test whether overexpression of Grain in all T4/T5 changes the optomotor response in the direction predicted by cell fate transformation. (Did these changes impaired postsynaptic connectivity of T4/T5 and could all of the b and c cells find the corresponding partners?)

We have recently started investigating how the transformations in T4/T5 morphology after TF code manipulation affect circuit function. For this, we are measuring electrophysiological responses of lobula plate tangential cells (LPTCs), which receive input from many hundreds of T4/T5 neurons. In this way, we have a direct readout of postsynaptic connectivity of T4/T5 neurons, whereas measuring the optomotor behaviour could lead to results that are much more ambiguous to interpret.

To date, we have only been able to record the visually evoked activity of LPTCs after expressing *grain-RNAi* in all T4/T5 neurons by means of the *R39H12-Gal4*, a condition that transforms T4/T5b,c into T4/T5a,d morphologies (**Fig. 6** and response to Reviewer 2). In wild-type animals, LPTCs with dendrites in lobula plate layer 1 (HS cells) receive direct, excitatory input from T4/T5a to depolarize to front-to-back motion, while they receive indirect, inhibitory input from T4/T5b to hyperpolarize to back-to-front motion (**Fig. R2A**). LPTCs with dendrites in lobula plate layer 4 (VS cells) receive direct, excitatory input from T4/T5d to depolarize to downward motion, while they receive indirect, inhibitory input from T4/T5c to hyperpolarize to upward motion (**Fig. R2B**) (Mauss et al., 2015). After transforming all T4/T5b,c into T4/T5a,d (*R39H12>grain-RNAi*), one could expect a normal or even enhanced depolarization of HS or VS cells to front-to-back or downward motion, and a strongly reduced or absent hyperpolarizing responses to back-to-front or upward motion. In this condition, indeed, we have found no hyperpolarizing responses in HS and VS cells. However, the depolarizing responses also seem to be strongly reduced (**Fig. R2A**) or absent (**Fig. R2B**). This occurs despite the fact that dendrites of HS and VS cells still innervate the lobula plate and overlap with T4/T5 axons (**Fig. R2C,D**). The preliminary results we obtained so far might be explained by a compromised connectivity between T4/T5 neurons and LPTCs upon *grain* knockdown in all T4/T5, which disrupts the normal layered structure of the lobula plate (**Fig. 6B**, **Fig. R2D**) and likely the overall organization of motion processing circuits in this neuropil. Therefore, more specific manipulations are required in order to test whether transformed T4/T5 neurons connect to new postsynaptic partners. For instance, one would need to transform very specifically a single T4 or T5 subtype to keep the layered organization of the lobula plate intact and to assess the connectivity between the transformed T4 or T5 subtype and its possible new LPTCs partners. This could be done by optogenetically activating only

© 2020. Published by The Company of Biologists under the terms of the Creative Commons Attribution License (https://creativecommons.org/licenses/by/4.0/).

the transformed T4 or T5 subtype while electrophysiologically recording from possible new LPTCs partners.

Furthermore, we are also interested in how the morphologically transformed T4/T5 neurons respond to moving stimuli. In a preliminary experiment, we have measured the visually evoked activity of T4/T5 neurons upon *grain* overexpression with the *R42F06-Gal4*, which transforms T4/T5a,d into T4/T5b,c. We found that each of the two layers of T4/T5 axons (**Fig. 5B**) selectively responds to either back-to-front or upward motion (**Fig. R3**), as expected if only T4/T5b,c are present (Maisak et al., 2013). In this experiment, however, one cannot distinguish the responses of T4/T5b,c that have remained T4/T5b,c from the responses of T4/T5a,d that have been transformed into T4/T5b,c neurons upon *grain* overexpression. Therefore, it remains possible that the observed activity arises exclusively from the former neurons while the later ones are not functional. To further test this, one would need to specifically record the visually evoked activity of T4/T5 subtypes that have been morphologically transformed, like the ones shown in **Fig. S6D,E**.

In summary, we consider that more experiments are needed to understand properly the functional implications of the transformations in T4/T5 morphology. This, however, will go far beyond the revision time. Therefore, we have decided not to include the preliminary data in the manuscript. We believe that this does not affect the quality of our study because this missing aspect is beyond its main scope, although it is very interesting for future studies.

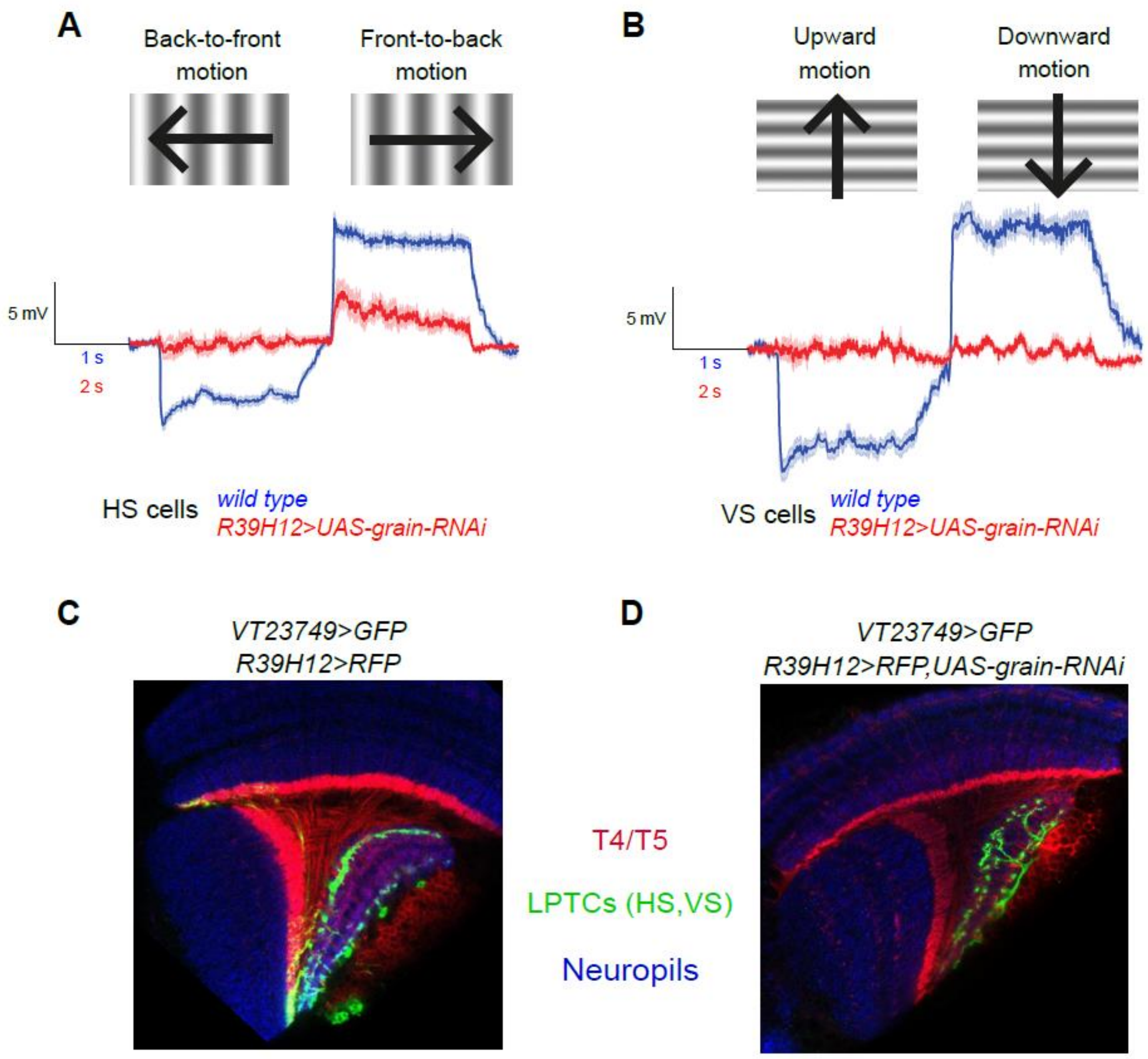

© 2020. Published by The Company of Biologists under the terms of the Creative Commons Attribution License (https://creativecommons.org/licenses/by/4.0/).

Figure R2. (A,B) Whole cell patch-clamp recordings from horizontally sensitive (HS) and vertically sensitive (VS) lobula plate tangential cells (LPTCs) upon 1Hz sine-wave visual grating motion along the four cardinal directions. Blue traces show fully opponent null direction and preferred direction responses of HS and VS cells in *Canton-S* wild-type flies (A: N=11 flies, n=19 cells; B: N=5 flies, n=7 cells). Red traces show strongly reduced responses of HS cells (N=5 flies, n=6 cells) and no responses of VS cells (N=5 flies, n=6 cells) upon *grain* knockdown in all T4/T5 neurons with the *R39H12-Gal4*. (C,D) Horizontal views of adult optic lobes showing the dendrites of LPTCs (HS and VS cells labelled with the *VT23749-LexA* line) in the presence of wild-type T4/T5 axons (C), and in the presence of T4/T5 axons with *grain* knockdown (D). Anti-Bruchpilot was used to label the neuropils.

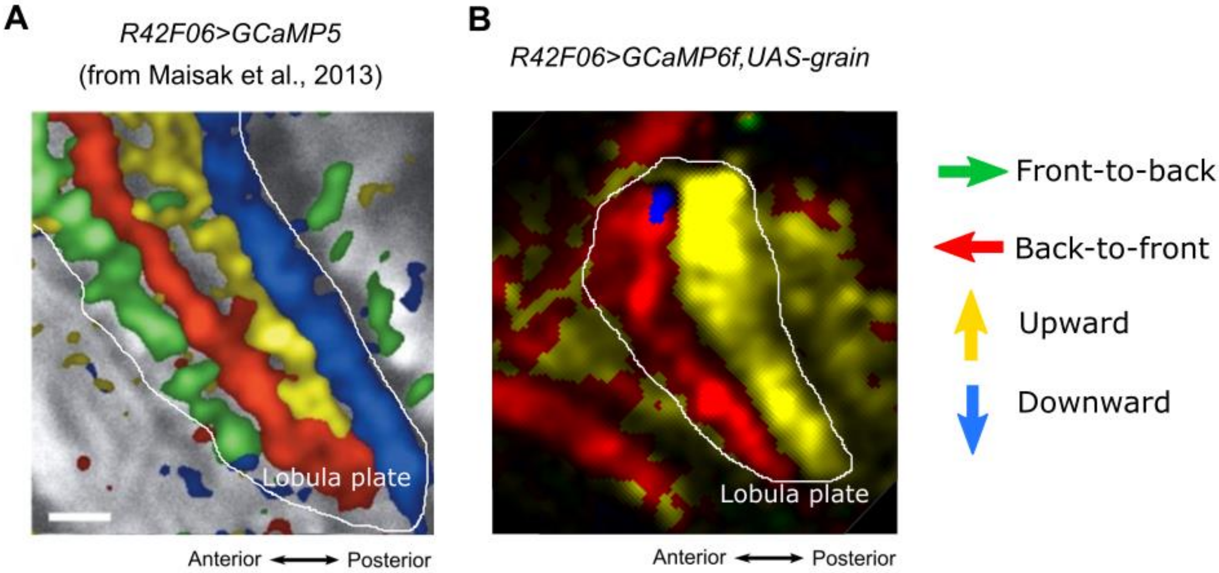

Figure R3. (A) Horizontal view of the lobula plate showing four layers of T4/T5 axons. Each layer is formed by axons of a single T4/T5 subtype that respond selectively to motion in one of the four cardinal directions (data from Maisak et al., 2013). (B) T4/T5 axons form only two layers in the lobula plate upon *grain* overexpression in all T4/T5 neurons with the *R42F06-Gal4* line. In this condition, responses to motion along four cardinal directions were optically recorded in T4/T5 axons expressing the calcium indicator GCaMP6f by using two-photon fluorescence microscopy. Back-to-front motion leads to activity in the anterior layer (in red, most likely T4/T5b axons) while upward motion leads to activity in the posterior layer (in yellow, most likely T4/T5c axons). T4/T5 axons did not respond to front-to-back or downward motion, normally detected by T4/T5a or T4/T5d subtypes. A particular colour was assigned to each pixel according to the stimulus direction during which it reached maximum value. Otherwise, it was assigned to background. The response strength of each pixel was coded as the saturation of that particular colour. These results represent the data obtained from a single fly averaged over several stimulus repetitions. Similar results were obtained from three other flies with the same genotype.

3) The authors suggest that selective expression of Grain in T4/T5 b and c subtypes is regulated by Notch signaling. The authors should test if Notch activation or inhibition in T4/T5 is sufficient to alter Grain. They could the use T4/T5 LexA driver (Strother 2017) combined with a LexAop-Notch-intra (Weinberger 2017) and examine the cell type expression of Grain-Gal4>UAS-GFP.

Each T4/T5-producing neuroblast divides to produce two ganglion mother cells, which divide once more to generate postmitotic T4/T5 neurons. During the neuroblast division, a first Notch binary cell-fate decision specifies T4/T5a versus T4/T5b (Omb$^-$ neuroblasts) and T4/T5c versus T4/T5d (Omb$^+$ neuroblasts). During the division of each ganglion mother cell, a second Notch binary cell-fate decision specifies T4 versus T5 identity. It has been proposed that transient Notch activity in these two distinct contexts instructs different gene expression programs (Pinto-Teixeira et al., 2018). In our discussion, we suggested that only the first Notch binary switch underlies the selective expression of *grain* in T4/T5b,c subtypes. This hypothesis is based on the patterns of *grain* expression in T4/T5 neurons (revealed in our work) and of Notch activity in their progenitors (Pinto-Teixeira et al., 2018),

© 2020. Published by The Company of Biologists under the terms of the Creative Commons Attribution License (https://creativecommons.org/licenses/by/4.0/).

as well as on previous work on the specification of another *Drosophila* neuronal type (Garces and Thor, 2006). We think that a detailed analysis of *grain* expression in T4/T5-producing neuroblasts, ganglion mother cells, and newborn T4/T5 neurons would be required to further investigate this, as well as an analysis of *grain* expression after manipulating the first Notch binary switch during neuroblast division. Unfortunately, the experiment suggested by the reviewer will not help towards this end because the *R42F06* enhancer fragment used to generate the T4/T5-LexA line (Strother et al., 2017) drives expression in postmitotic T4/T5 neurons but not in T4/T5-producing neuroblasts and ganglion mother cells (**Fig. S4A**).
Although we agree that it would be very exciting to investigate how the T4/T5 subtype-specific expression of *grain* is achieved during development, we believe that this goes beyond the scope of this paper and the amount of work required for this cannot be completed during the revision time. Therefore, investigations on this topic are better suited for future studies.

Second decision letter

MS ID#: DEVELOP/2019/186296

MS TITLE: A combinatorial code of transcription factors specifies subtypes of visual motion-sensing neurons in Drosophila

AUTHORS: Nikolai Hörmann, Tabea Schilling, Aicha Haji Ali, Etienne Serbe, Christian Mayer, Alexander Borst, and Jesús Pujol-Martí

I have now received the reports of two of the referees who reviewed the earlier version of your manuscript and I have reached a decision. The referees' comments are appended below, or you can access them online: please go to BenchPress and click on the 'Manuscripts with Decisions' queue in the Author Area.

The reviewers' evaluation is positive and we would like to publish a revised manuscript in Development, provided that you satisfactorily address the remaining minor comment of referee 3. Please attend to this comment in your revised manuscript and in your point-by-point response. If you do not agree with it, explain clearly why this is so.

Reviewer 2

Advance summary and potential significance to field

See my previous review

Comments for the author

Well done!

Reviewer 3

Advance summary and potential significance to field

The main question of this study is what are the molecular basis of neuronal cell-type specification? As a model, the authors used T4/T5 neurons of Drosophila visual system, which are well described anatomically and functionally. These cells derive from the same progenitor pool, although during development they form eight distinct cell-types with unique innervation position in Lobula plate and dendritic arbor direction. These morphological features also determine the specific function of each cell-type in fly motion detection vision. In this work Hoermann et. al. wanted to understand how these unique features could be acquired.

© 2020. Published by The Company of Biologists under the terms of the Creative Commons Attribution License (https://creativecommons.org/licenses/by/4.0/).

To answer the main question of the study, the authors first determined a temporal window in which dendritic branches are formed. Subsequently they performed scRNA-seq and compared transcriptomes of T4/T5 cell-types in different developmental stages. After detailed analysis they found a combinatorial regulatory code of five transcriptional factors that regulates development of T4/T5 dendrite orientation and axon projection patterns.
In my opinion the strong part of the paper is an accurate analysis of transcriptional profiles from scRNA-seq data, in which they found a range of differentially expressed genes within eight T4/T5 clusters in different time points, which could play variety of roles in cells development. Additionally, it was clearly shown that a minimal transcription factor code of five TFs could reflect identity of all eight cell types. The phenotype observed upon Grain missexpression is a strong indicator that manipulating this code changes cell-type identity.
In general, I find this work original, interesting and well done. The work is well illustrated, the figures are structured and easy to interpret.

Comments for the author

The authors have done a very good job revising the paper and explaining why certain issue should not be directly addressed. I thought their answers were fair, and therefore have no further experimental comments. I would only suggest that they the down the conclusions regarding the role of Notch in Grain regulation, as they can't show experimentally that Notch does regulate Grain expression (line 291-294)

Second revision

Author response to reviewers' comments

Reviewer 3 Comments for the author
The authors have done a very good job revising the paper and explaining why certain issue should not be directly addressed. I thought their answers were fair, and therefore have no further experimental comments.
I would only suggest that they the down the conclusions regarding the role of Notch in Grain regulation, as they can't show experimentally that Notch does regulate Grain expression (line 291-294).

Response for Reviewer 3:
We thank the reviewer for his/her positive feedback. In our previous response to reviews, we forgot to mention that in the last version of the manuscript we did tone down this part of the discussion (lines 308-312), highlighting that "Future studies will need to investigate how the T4/T5 subtype-specific expression of *grain*, as well as *ab* and *pros*, is achieved during development."

Third decision letter

MS ID#: DEVELOP/2019/186296

MS TITLE: A combinatorial code of transcription factors specifies subtypes of visual motion-sensing neurons in Drosophila

AUTHORS: Nikolai Hörmann, Tabea Schilling, Aicha Haji Ali, Etienne Serbe, Christian Mayer, Alexander Borst, and Jesús Pujol-Martí
ARTICLE TYPE: Research Article

I am delighted to tell you that your manuscript has been accepted for publication in Development, pending our standard ethics checks.

© 2020. Published by The Company of Biologists under the terms of the Creative Commons Attribution License (https://creativecommons.org/licenses/by/4.0/).
